# Shp1 phosphatase regulates CXCR2 protein stability and IL8-mediated invasiveness in breast cancer

**DOI:** 10.1038/s41419-026-08516-4

**Published:** 2026-03-02

**Authors:** Marcello Monti, Pier Giorgio Amendola, Angela Filograna, Sabrina Gargiulo, Marcello Allegretti, Daniela Corda, Alessia Varone

**Affiliations:** 1 Institute of Endotypes in Oncology, Metabolism and Immunology “G. Salvatore”, National Research Council, Naples, Italy; 2 Research & Early Development, Dompé Farmaceutici SpA, L’Aquila, Italy

**Keywords:** Tumour-suppressor proteins, Extracellular signalling molecules

## Abstract

Shp1 is a cytosolic tyrosine phosphatase generally associated with antitumor effects through the inhibition of tyrosine kinase signaling. Herein, we shown that genetic and pharmacological inhibition of Shp1 in breast cancer cells induces accelerated cell migration and promotes a more invasive phenotype. Furthermore, we found that interleukin-8 (IL8), a chemokine with multiple pro-tumorigenic roles within the tumor microenvironment, directly modulates Shp1 activity. In breast cancer, IL8 elicits its functions through the binding to the CXCR2 receptor with the subsequent modulation of several intracellular signaling pathways. We show that in breast MCF7 cells, IL8 induces the PKC-mediated phosphorylation of Shp1 at Ser591, diminishing its enzymatic activity and impairing the dephosphorylation of PP2A; this enhances CXCR2 phosphorylation and alters receptor trafficking by promoting ubiquitination and degradation of CXCR2. This feedback mechanism limits IL8 signaling revealing a previously unrecognized mechanism of receptor turnover and signal attenuation. In addition, we found that Shp1-mediated regulation of CXCR2 directly influences IL8-driven invasiveness in a subtype-specific manner, affecting luminal and triple-negative breast cancer (TNBC) cells but not HER2-positive ones. Transcriptomic and pathway analyses further support Shp1 involvement in cytokine and GPCR signaling, particularly in TNBC, where its downregulation correlates with reduced survival and higher IL8 levels. Taken together, our findings elucidate a novel mechanism of IL8 signaling and identify Shp1 as a promising therapeutic target, highlighting the potential of modulating the CXCR2–Shp1 axis to limit invasiveness and metastasis in aggressive breast cancer subtypes, particularly TNBC.

## Introduction

According to the 2024 cancer statistics [1], breast cancer remains a leading cause of cancer-related deaths among women aged 20–59, with rising incidence and persistent global impact [2]. Understanding the molecular mechanisms of carcinogenesis is therefore essential for advancing treatment and reducing mortality.

Accumulating evidence, including our studies, revealed the involvement of the tyrosine phosphatase Shp1 (Src homology 2 domain-containing phosphatase 1) in several cancers [3–5], including breast cancer, where it acts as a tumor suppressor and enhances sensitivity to chemotherapeutic treatments [6–8].

Shp1 regulates cell signaling through a complex mechanism involving autoinhibition and phosphorylation events [9]. It consists of two N-terminal SH2 domains, a catalytic domain, and a C-terminal tail with phosphorylation sites. SRC-kinase-dependent (Tyr536/Tyr564) and PKC-dependent (Ser591) modifications, respectively, enhance and inhibit Shp1 activity [10]. These phosphorylations affect Shp1 activity, stability, localization, and interactions along the pathway, enabling tight regulation of signaling as a general key-negative regulator [9].

Shp1 loss of function is indeed implicated in the onset and progression of various malignancies. For instance, hypermethylation of Shp1 promoter, common in leukemias and lymphomas, reduces protein expression, enhancing tyrosine kinase signaling [11, 12]. In solid cancers, Shp1 loss correlates with poor prognosis and hyperactivation of oncogenic pathways such as JAK/STAT, NF-κB, and Akt pathways, further supporting its tumor-suppressor role [3, 13]. In breast cancer, Shp1 inhibits proliferation mainly through the modulation of the JAK/STAT3 pathway [14–16]. Additionally, Shp1 directly interacts with EGFR and HER2, suppressing downstream pathways associated with both cell cycle and EMT [6, 7]. Beyond these receptors, Shp1 also targets other membrane receptors such as VEGFR, PDGFR [17, 18], and cytokine receptors like G-CSF, IL2, and IL3 [19, 20]. Through its wide range of targets, Shp1 regulates both cancer cells and the tumor microenvironment, influencing stromal and immune interactions that drive cancer progression.

Tumor microenvironment is shaped by various factors, including cytokines, small signaling molecules produced by both immune and tumor-associated cells. Cancer cells use cytokines to boost growth, spread, and drug resistance [21]. Cytokines also recruit and activate nearby cells, promote angiogenesis, and aid immune evasion [22]. Interleukin-8 (IL8) is a cytokine upregulated in many cancers, where it promotes tumor growth, angiogenesis, survival, and immune modulation [23]. This is achieved through IL8 binding to the G-protein-coupled receptors (GPCRs) CXCR1/2, which modulate several intracellular signaling [24], including the PLC/PKC, PLD, PI3K/Akt, Ras/Erk, and the JAK2/STAT3 signaling pathways [25]. Given the variety and complexity of these pathways, IL8 significantly impact on gene expression, cytoskeleton organization, and protein modifications [26, 27].

In breast, IL8 expression has been linked to the metastatic potential of cancer cells, as high IL8 levels enhance cell invasiveness and promote EMT, stem cell-like traits, and tumor initiation [23, 28–32]. Importantly, blocking IL8 or IL8 receptors significantly reduces invasiveness and decreases cancer stem cells in vitro and in vivo [33, 34].

Despite the central role of IL8-CXCR2 signaling in breast cancer progression, its regulation by Shp1 has not been investigated. Yet, Shp1 is known to modulate the phosphorylation state, activity, and downstream signaling of multiple receptor tyrosine kinases and cytokine receptors. The absence of data on Shp1-CXCR2 interactions, therefore, represents a critical knowledge gap, raising the possibility that Shp1 may also contribute to IL8-driven tumor behavior. Building on this rationale, our findings demonstrate that Shp1 is critical for IL8-driven cancer progression, promoting tumor invasiveness by regulating CXCR2 degradation via ubiquitination. Moreover, the identification of Shp1 as a novel component of the CXCR2-IL8 pathway in a specific set of breast cancers may significantly impact future research on immune-inflammatory responses and cancer biology.

## Results

### Shp1 negatively affects migration and invasion of MCF7 cells

We investigated the role of Shp1 in breast cancer progression using the human breast adenocarcinoma cell line MCF7 as a model system. Shp1 regulation of cell motility in breast cancer was previously reported in overexpression studies [6, 7]; here we examined its contribution using two alternative approaches: the CRISPR-generated Shp1 knock-out MCF7 cells (Shp1-KO) and the chemical inhibition with a well-established Shp1 inhibitor, sodium stibogluconate (SSG).

**Fig. 1:**
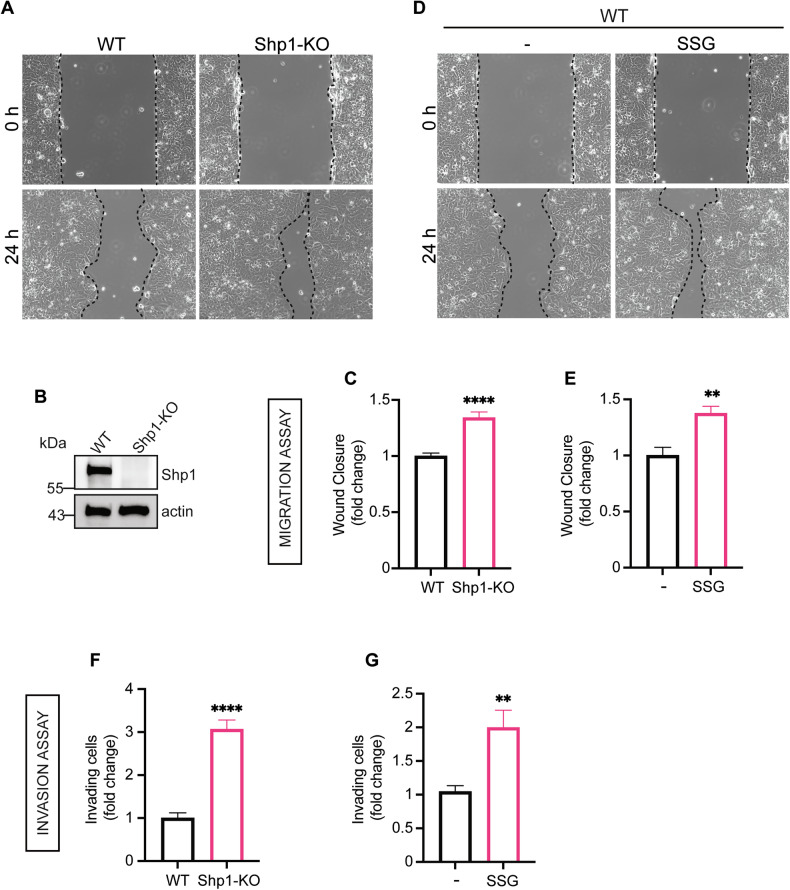
Shp1 inhibition induces MCF7 cell migration and invasion. **A** Representative images of wound-closure assays of MCF7 (WT) and MCF7 Shp1-KO (Shp1-KO) cells. **B** Representative western blot using anti-Shp1 specific antibody in WT or KO Shp1 MCF7 cells. β-actin was used as loading control. Data are representative of three independent experiments. Molecular weight standards (kDa) are indicated on the left of each panel. **C** Quantification of the extent of wound closure calculated by analyzing the scratched area covered by cells in (**A**) after 24 h using ImageJ software. **D** Representative images of wound closure assays of MCF7 cells (WT) after 24 h of treatment with PBS (vehicle control; −) or SSG (10 μM). **E** Quantification of the extent of wound closure calculated by analyzing the scratched area covered by cells in (**D**) after 24 h using ImageJ software. **F**, **G** Quantification of Matrigel invasion assays of MCF7 and MCF7 Shp1-KO cell lines, respectively, treated as in (**A**, **D**). Invasion was quantified by crystal violet staining, elution of invading cells, and data are presented as fold increase in invading cells. Data are mean ± SD of three independent experiments performed in triplicate. ****P < 0.0001, **P < 0.005 versus WT cells or vehicle control (Student’s t-tests).

We analyzed the effects of Shp1 inhibition in cell migration and invasion by wound-healing and transwell-matrigel invasion assays (see “Materials and methods”). Shp1-KO cells show a significant increase in the migration rate with respect to the wild-type MCF7 cells, with an increase of 40% after 24 h (Fig. 1A–C). Similarly, wound-healing assays on MCF7 cells exposed to SSG showed a similar increased cell migration (Fig. 1D, E). The transwell-invasion assay also demonstrated a marked increase in the average number of cells penetrating the Matrigel in both Shp1-KO and SSG-treated MCF7 cells (a 3-fold and a 2-fold increase, respectively) (Fig. 1F, G). Comparable results were obtained upon transient silencing of Shp1 using siRNAs and in an independent Shp1 knock-out clone (Shp1-KO cl.2), both of which showed increased migratory and invasive capacity compared with controls (Figs. S1A, C, E and S2D).

We then analyzed public-gene expression datasets linked to clinical data to assess Shp1 impact on breast-cancer patients’ survival. The analysis of the Cancer Genome Atlas (TCGA) and the breast invasive carcinoma (BRCA) dataset via Gene Expression Profiling Interactive Analysis (GEPIA) revealed that a lower Shp1 expression is linked to poorer overall survival (Fig. S2A). Similarly, results from the Kaplan–Meier Plotter database confirmed that reduced Shp1 correlates with shorter relapse-free survival (*p* = 0.018; Fig. S2B), suggesting a worse prognosis.

Overall, these findings indicate that Shp1 acts as a negative regulator of cell migration and invasion, with its catalytic activity having a crucial role in these processes. This is further supported by the observation that low Shp1 expression levels are associated with a more aggressive behavior in breast cancer patients.

### Shp1 inhibition suppresses IL8 function in MCF7 cells

Breast cancer cells secrete IL8, which supports cancer progression, promoting proliferation, angiogenesis, and metastasis [35–37]. Considering the Shp1 role in cytokine signaling, we explored its interaction with IL8 in promoting cancer invasiveness.

**Fig. 2:**
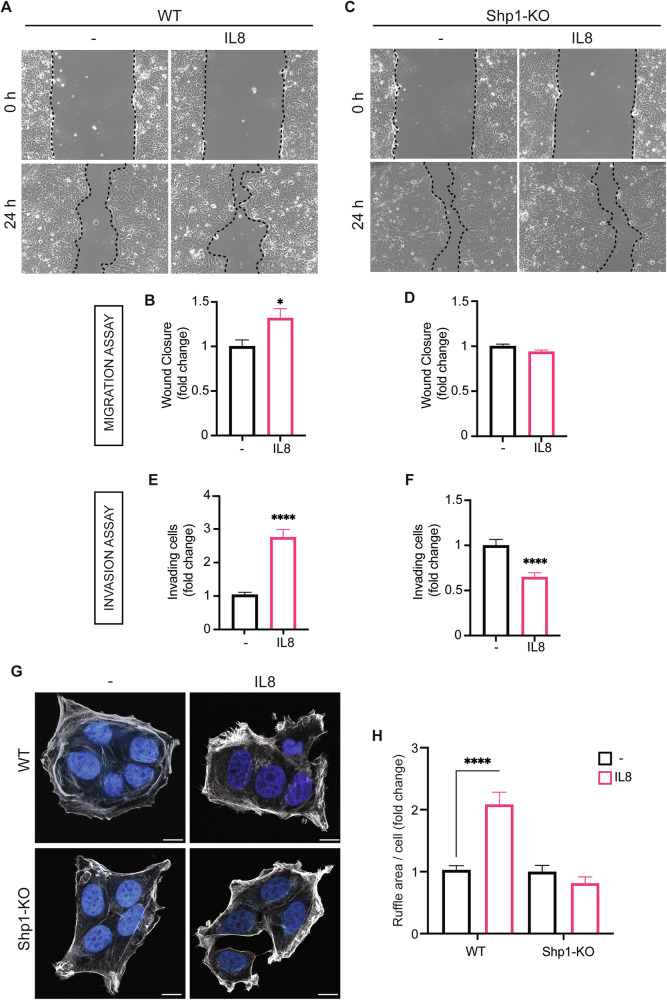
Shp1 inhibition impairs IL8-induced MCF7 cell migration and invasion. **A** Representative images of wound closure assays of MCF7 cells (WT) after 24 h of treatment with PBS (vehicle control; −) or IL8 (250 ng/ml). **B** Quantification of the extent of wound closure calculated by analyzing the scratched area covered by cells in (**A**) after 24 h using ImageJ software. **C** Representative images of wound closure assays of Shp1-KO cells (Shp1-KO) after 24 h of treatment with PBS (vehicle control; −) or IL8 (250 ng/ml). **D** Quantification of the extent of wound closure calculated by analyzing the scratched area covered by cells in (**C**) after 24 h using ImageJ software. **E** Quantification of Matrigel invasion assays of MCF7 cells treated as in (**A**). **F** Quantification of Matrigel invasion assays of MCF7 Shp1-KO cells treated as in (**C**). Invasion was quantified by crystal violet staining, elution of invading cells, and data are presented as fold increase in invading cells. Data are mean ± SD of three independent experiments performed in triplicate. ****P < 0.0001, *P < 0.05 versus vehicle control (Student’s t-tests). **G** Representative images of MCF7 and MCF7 Shp1-KO cells treated with PBS (vehicle control; −) or IL8 (250 ng/ml) for 1 h (as indicated). Cells were fixed and stained with phalloidin (gray) and Hoechst 33342 (blue). Scale bars, 10 μm. **H** Quantification of ruffle area/cell (expressed as percentage of control) of cells in (**G**) (see “Materials and methods”). Data are mean ± SD of three independent experiments. ****P < 0.0001 versus Ctrl (WT cells treated with vehicle control) (Student’s t-tests).

IL8-induced MCF7 cell invasiveness was tested under Shp1 inhibition using wound-healing and invasion assays. IL8 treatment for 24 h increased the migration and invasion of MCF7 cells (by 30% and 170%, respectively; Fig. 2A, B, E), whereas in Shp1-KO cells, these IL8 pro-invasive effects were almost completely abolished (Fig. 2C, D, F). Notably, under conditions of Shp1 inhibition, IL8 not only failed to promote invasiveness but actually reduced tumor cell invasion (Fig. 2F), indicating that IL8 may exert opposing effects depending on the presence of functional Shp1. Consistent results were observed in cells transiently transfected with Shp1 siRNAs and in Shp1-KO cl.2, further confirming that the inhibitory effect of IL8 on invasiveness requires functional Shp1 (Figs. S1E, F and S2D).

Cell migration depends on dynamic remodeling of the actin cytoskeleton, including the formation of F-actin-rich ruffles that initiate motility [38]. We therefore measured the average ruffle area per cell in fixed samples of wild-type and Shp1-KO cells treated with IL8 for 1 h, stained for F-actin, and analyzed for cytoskeletal changes (see “Materials and methods”). In control cells, IL8 triggered membrane ruffling and actin reorganization, indicating higher motility, while had no effect in Shp1-KO cells (Fig. 2G), as confirmed by actin staining quantification (Fig. 2H) [39].

These findings, consistently with the wound-healing and invasion results, lead to the conclusion that IL8 promotes breast cancer cell migration and invasion through Shp1.

### IL8 inhibits Shp1 enzymatic activity

**Fig. 3:**
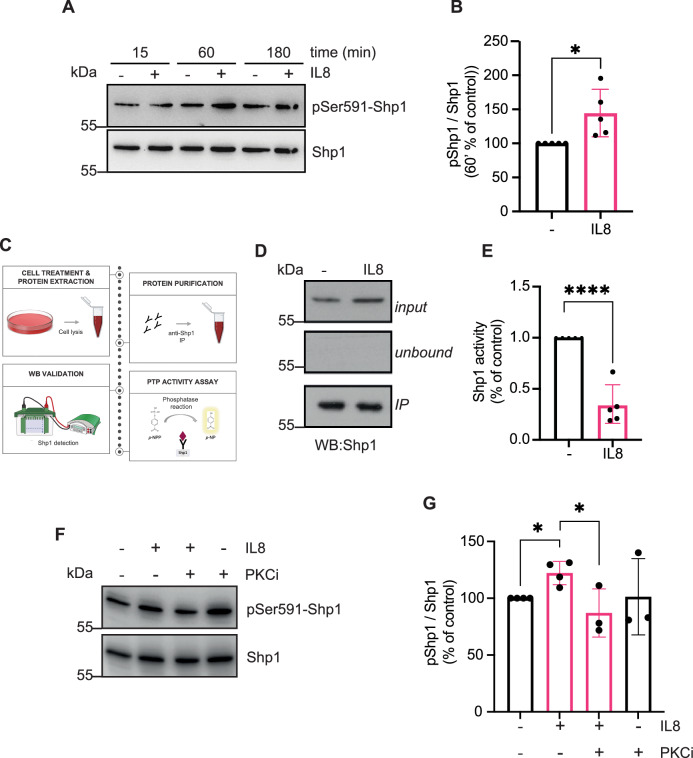
IL8 affects Shp1 phosphorylation and enzymatic activity in MCF7 cells. **A** Representative western blot using anti-phosphoserine 591 in Shp1 (pSer591-Shp1) specific antibody in MCF7 cells treated with PBS (vehicle control; −) or IL8 (250 ng/ml; +) for the indicated times. Total Shp1 was used as loading controls. Data are representative of five independent experiments. Molecular weight standards (kDa) are indicated on the left of each panel. **B** Densitometric quantification of the blot in (**A**) (60 min). Data are mean ± SD of five independent experiments. *P < 0.05 versus control. **C** Schematic representation of the experimental workflow for Shp1 immunoprecipitation and phosphatase activity assay. **D** Representative immunoprecipitated Shp1 fraction (IP) from MCF7 cells treated with PBS (vehicle control; −) or IL8 (250 ng/ml) for 60 min (as indicated). The top panels show the input and the unbound fractions. Molecular weight standards (kDa) are indicated on the left of each panel. **E** Quantification of enzymatic activity of Shp1 immunoprecipitated as in (**C**) (see “Materials and methods”). Data are mean ± SD of five independent experiments. ****P < 0.0001 versus ctrl (WT cells treated with vehicle control) (Student’s t-tests). **F** Representative western blot using anti-phosphoserine 591 in Shp1 (pSer591-Shp1) specific antibody in MCF7 cells treated with PBS (vehicle control; −) or IL8 (250 ng/ml; +) for 1 h in absence or presence of PKCi (1 μM). Total Shp1 and GAPDH were used as loading controls. Data are representative of at least three independent experiments. Molecular weight standards (kDa) are indicated on the left of each panel. **G** Densitometric quantification of the blot in (**F**). Data are mean ± SD of at least three independent experiments. *P < 0.05 versus ctrl (vehicle control; −).

To elucidate how Shp1 drives IL8-induced cancer invasion, we analyzed its phosphorylation state following IL8 challenge. Serine phosphorylation inhibits Shp1 activity [40], while PKC, a downstream target of CXCR1/2, is known to regulate Shp1 phosphorylating Ser591 [40–42]. Thus, this modification was analyzed in cells treated with IL8 for different times (see “Materials and methods”); an increase in Shp1-Ser591 phosphorylation was detected at 60 min (Fig. 3A, B). MCF7 cells were then treated with IL8 for 60 min and Shp1 enzymatic activity measured using p-Nitrophenyl Phosphate (pNPP) as a substrate (see “Materials and methods”; Fig. 3C). This revealed a Shp1-reduced enzymatic activity by ~60% (Fig. 3D, E), indicating that Ser591-phosphorylation significantly impairs Shp1 catalytic function. To further analyze Shp1 regulation, IL8-stimulated MCF7 cells treated with the generic PKC inhibitor Bisindolylmaleimide I (PKCi) revealed a decrease of Ser591 phosphorylation, confirming PKC contribution in Shp1 regulation (Fig. 3F, G).

Together, these data demonstrate that IL8 significantly inhibits Shp1 enzymatic activity, an effect exerted through the PKC-mediated phosphorylation of the Ser591 residue.

### Shp1 regulates CXCR2 receptor at the post-translational level

Shp1 role in IL8-driven breast cancer invasiveness was explored analyzing CXCR2 signaling. Like other GPCRs, CXCR2 activity depends on its phosphorylation; when activated by IL8, serine phosphorylation by GRKs causes the receptor to be internalized into early endosomes, where PP2A dephosphorylates it, allowing recycling of the receptor [43]. As reported in leukemia cells, Shp1 can activate PP2A by dephosphorylating its Tyr307 [44, 45]. Based on this, we investigated how the Shp1, PP2A, and IL8-CXCR2 pathway behaves in breast cancer.

**Fig. 4:**
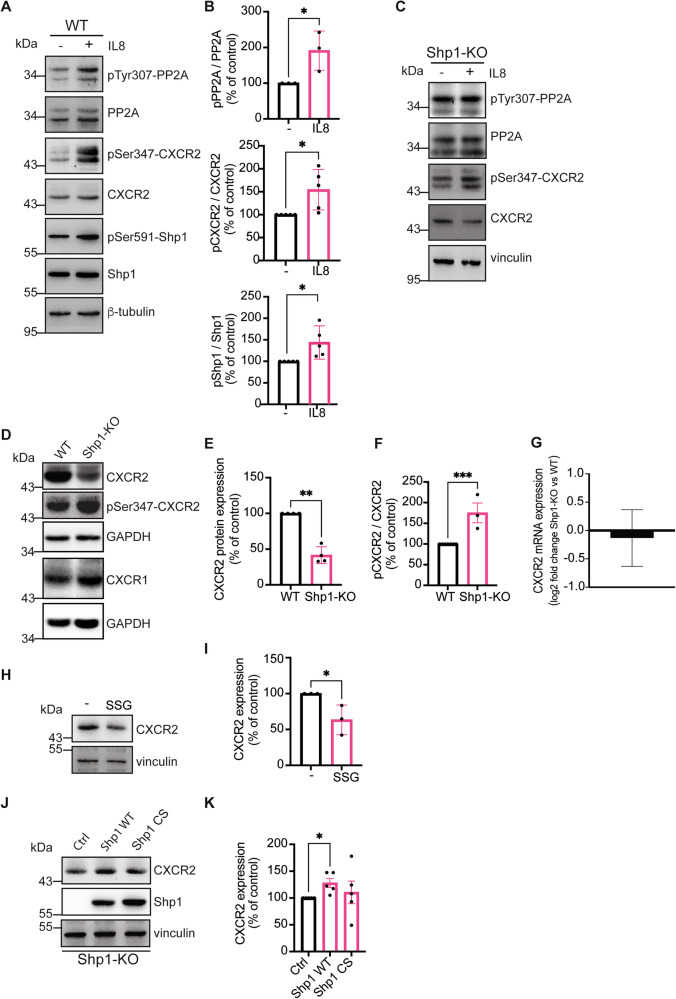
Shp1 controls IL8-induced CXCR2 phosphorylation and regulates CXCR2 protein levels. Representative western blot using anti-phosphotyrosine 307 in PP2A (pTyr307-PP2A), anti-phosphoserine 347 in CXCR2 (pSer347-CXCR2), and anti-phosphoserine 591 in Shp1 (pSer591-Shp1) specific antibodies in MCF7 (**A**) and MCF7 Shp1-KO (**C**) cells treated with PBS (vehicle control; −) or IL8 (250 ng/ml; +) for 1 h. Total CXCR2, PP2A, Shp1, β-tubulin, and vinculin were used as loading controls. Data are representative of at least three independent experiments. Molecular weight standards (kDa) are indicated on the left of each panel. **B** Densitometric quantification of the blot in (**A**). Data are mean ± SD of at least three independent experiments. *P < 0.05 versus ctrl. **D** Representative western blot using anti-CXCR2, anti-phosphoserine 347 in CXCR2 (pSer347-CXCR2) and anti-CXCR1 specific antibodies in cell lysates from MCF7 WT and Shp1-KO cells. GAPDH was used as loading control. Molecular weight standards (kDa) are indicated on the left of each panel. Data are representative of three independent experiments. **E**, **F** Densitometric quantification of the blots in (**D**). ***P < 0.0005; **P < 0.005 versus WT cells (Student’s t-tests). **G** Analysis by qRT-PCR of mRNA levels of CXCR2 in MCF7 WT and Shp1-KO cells. β-actin was used as housekeeping gene. Data are expressed as log2 fold change of Shp1-KO *versus* WT cells. Data are means ± SD of three independent experiments performed in triplicate. **H** Representative western blot using anti-CXCR2 specific antibody in MCF7 cells treated with PBS (vehicle control; −) or SSG (10 μM) for 48 h. Vinculin was used as loading control. Data are representative of three independent experiments. Molecular weight standards (kDa) are indicated on the left of each panel. **I** Densitometric quantification of the blot in (**H**). *P < 0.05 versus Ctrl (Student’s t-tests). **J** Representative western blot using anti-CXCR2 and anti-Shp1 specific antibodies in MCF7 Shp1-KO cells transfected with Shp1 WT and Shp1 C455S mutant. The expression levels of Shp1 WT and Shp1 C455S indicate comparable amounts of proteins. Vinculin was used as loading control. Data are representative of five independent experiments. Molecular weight standards (kDa) are indicated on the left of each panel. **K** Densitometric quantification of the blot in (**J**). *P < 0.05 versus Ctrl (untransfected cells) (Student’s t-tests).

We show that in MCF7 cells, IL8 stimulation increases the inhibitory phosphorylation of PP2A at Tyr307 (see “Materials and methods”, Fig. 4A, B), suggesting a potential reduction of its activity. This was confirmed by examining the CXCR2 phosphorylation, a known substrate of PP2A (Fig. 4A, B). Indeed, IL8 treatment significantly increased the phosphorylation of CXCR2 at Ser347, that was minimal in untreated cells (Fig. 4A, B), supporting a mechanism involving impaired dephosphorylation rather than a direct modification.

This increase in phosphorylation of both PP2A and CXCR2 coincided with Shp1 phosphorylation at Ser591 (Fig. 4A, B), which, as shown previously, inhibits Shp1 activity (Fig. 3E). Importantly, in Shp1-KO cells, IL8 did not affect PP2A or CXCR2 phosphorylation, in line with the notion that Shp1 is necessary for this pathway (Fig. 4C).

A model deriving from this data envision that Shp1 and PP2A cooperate in regulating CXCR2 phosphorylation. However, upon IL8 stimulation, Shp1 becomes inactive, leading to PP2A inhibition and sustained CXCR2 phosphorylation (see model in Fig. 6C). This may impair receptor recycling with an impact on the CXCR2 final fate, directing it either towards recycling or degradation. Thus, while the data show a direct connection among Shp1, PP2A, and CXCR2, the causal relationship in this cascade remains to be established.

In western blot analyses, we observed that the expression level of CXCR2 was significantly higher in wild-type compared to Shp1-KO cells. Conversely, the levels of phosphorylated CXCR2 were elevated in Shp1-KO cells relative to wild-type cells (Fig. 4D, F). This reduction was not accompanied by changes at the mRNA level, as shown by qRT-PCR analysis (Fig. 4G; see “Materials and methods”).

These observations led us to consider that Shp1 may be involved in the regulation of CXCR2 expression levels, likely through the control of its phosphorylation. Indeed, in MCF7 wild-type cells Shp1 activity inhibition by SSG, resulted in a ~50% reduction in CXCR2 protein levels (Fig. 4H, I; see “Materials and methods”). Additionally, rescue experiments in which Shp1 was reintroduced in Shp1-KO cells showed that protein levels were recovered only with the active form of the protein (Shp1-WT) and not with a catalytically inactive mutant (Shp1-CS) (Fig. 4J, K).

Taken together, these results indicate that Shp1 plays a role in regulating CXCR2 at the post-translational level, with its enzymatic activity being important for the increased stability of CXCR2.

### Shp1 regulates the ubiquitin-proteasome degradation of CXCR2

Several studies have shown that CXCR2 fate depends on the duration of ligand stimulation: after 60 min, it recycles back to the plasma membrane, but prolonged stimulation causes degradation [43, 46]. Considering our data on the effect of Shp1 on CXCR2 stability, we investigated whether Shp1 also regulates ligand-induced CXCR2 degradation.

**Fig. 5:**
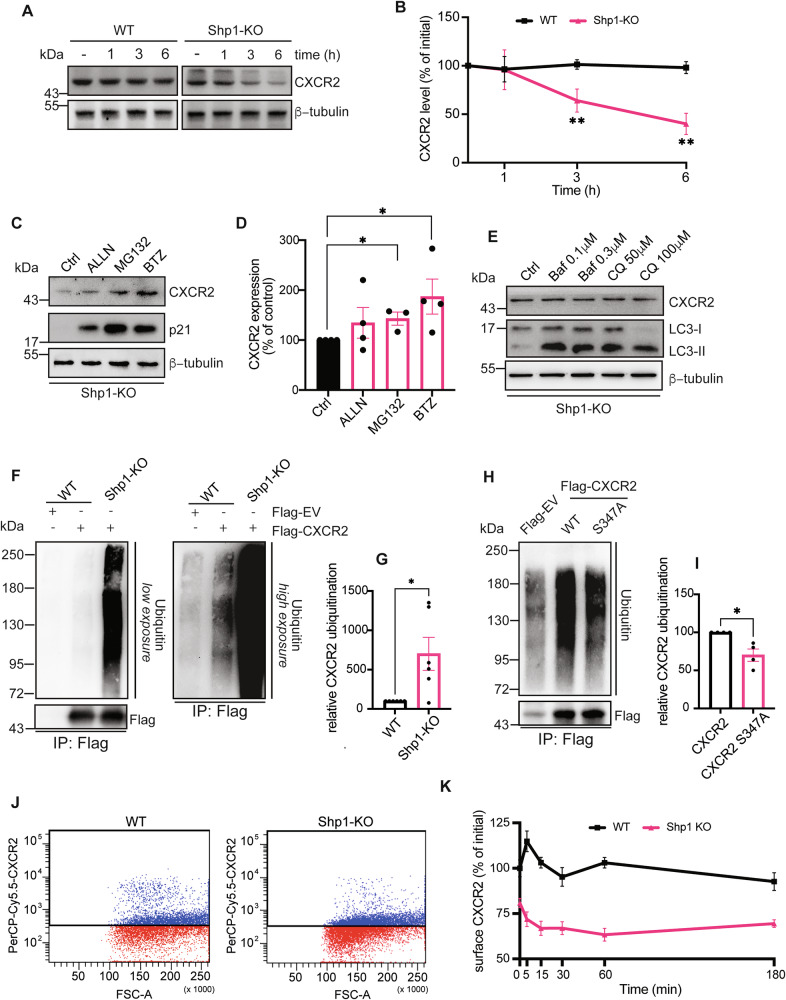
Shp1 regulates the ubiquitin-proteasome proteolysis of CXCR2. **A** Representative western blot using anti-CXCR2 specific antibody in MCF7 (WT) and MCF7 Shp1-KO (Shp1-KO) cells treated with CHX (25 μg/ml) and IL8 (250 ng/ml) for the indicated time points (see “Materials and methods”). β-tubulin was used as loading control. Data are representative of at least five independent experiments. Molecular weight standards (kDa) are indicated on the left of each panel. **B** Densitometric quantification of the blot in (**A**). **P < 0.005 versus time zero (Student’s t-tests). Representative western blot using anti-CXCR2 specific antibody in MCF7 Shp1-KO cells treated with DMSO (vehicle control; Ctrl), 20 μM ALLN, 25 μM MG132, or 1 μM Bortezomib for 4 h (**C**) or with DMSO (vehicle control; Ctrl), 0.1 and 0.3 μM Bafilomycin A1 or 50 and 100 μM Chloroquine for 4 h (**E**). The expression levels of p21 and LC3 I-II were monitored to evaluate inhibitors efficacy. β-tubulin was used as loading control. Data are representative of at least three independent experiments. Molecular weight standards (kDa) are indicated on the left of each panel. **D** Densitometric quantification of the blot in (**C**). *P < 0.05 versus Ctrl (Student’s t-tests). **F** Representative western blot using anti-ubiquitin antibody in the precipitate CXCR2 fraction (IP) from MCF7 cells transfected with an empty 3xFlag-vector or with 3xFlag-CXCR2 and from MCF7 Shp1-KO cells transfected with 3xFlag-CXCR2. The cells were treated with Bortezomib (1 μM) for 16 h, and then the lysates were immunoprecipitated using anti-Flag beads (see “Materials and methods”). Ubiquitin staining of 3xFlag-CXCR2 is shown with two different exposure times (low and high) for better interpretation. The protein levels of total 3xFlag-CXCR2 were detected with anti-Flag antibody and indicate comparable amounts of transfected protein (lower panel). Data are representative of six independent experiments. Molecular weight standards (kDa) are indicated on the left of each panel. **G** Quantification of ubiquitinylated receptor as in (**F**). *P < 0.05 versus WT (MCF7 cells transfected with 3xFlag-CXCR2) (Student’s t-tests). **H** Representative western blot using anti-ubiquitin antibody in the precipitate CXCR2 fraction (IP) from MCF7 cells transfected with an empty 3xFlag-vector or with 3xFlag-CXCR2 (WT) or with 3xFlag-CXCR2 S347A mutant (S347A). The cells were treated with Bortezomib (1 μM) for 16 h, and then the lysates were immunoprecipitated using anti-Flag beads (see “Materials and methods”). The protein levels of total 3xFlag-CXCR2 were detected with anti-Flag antibody and indicate comparable amounts of transfected proteins (lower panel). Data are representative of four independent experiments. Molecular weight standards (kDa) are indicated on the left of each panel. **I** Quantification of ubiquitinylated receptor as in (**H**). *P < 0.05 versus CXCR2 (MCF7 cells transfected with 3xFlag-CXCR2) (Student’s t-tests). **J** Representative flow cytometry analysis of CXCR2-surface expression of MCF7 (WT) and MCF7 Shp1-KO (Shp1-KO) cells (as indicated) stained with PerCP-Cy5.5-anti-CXCR2 antibody. The gate was set according to the negative control in the different cell lines. Data are representative of at least four independent experiments. **K** Quantification of surface expression of CXCR2 in MCF7 WT and Shp1-KO cells treated with IL8 (250 ng/ml) for the indicated time points and analyzed by flow cytometry (see “Materials and methods”). Data are expressed as percentages of control (untreated MCF7 WT cells). Data are mean ± SE of at least four independent experiments.

In MCF7 wild-type cells treated with IL8 and cycloheximide, CXCR2 levels remained relatively stable, with no major changes observed after 6 h (Fig. 5A, B; see “Materials and methods”). In contrast, Shp1-KO cells showed faster degradation with 60% and 40% remaining at 3 and 6 h, respectively.

Intracellular protein degradation occurs via either lysosomal or ubiquitin-proteasome systems. To determine which mechanism is involved in CXCR2 degradation, Shp1-KO cells were analyzed by western blot after treatment with the lysosome inhibitors chloroquine and bafilomycin A1 or the proteasome inhibitors ALLN, MG132, and Bortezomib (see “Materials and methods”). ALLN, MG132, and Bortezomib led to a clear increase in CXCR2 protein levels (Fig. 5C, D), while chloroquine and bafilomycin A1 had no effect (Fig. 5E). To confirm the effectiveness of the inhibitors, we monitored the levels of p21 and the lipidated form of LC3 (LC3-II) as positive controls for proteasome and lysosome inhibition, respectively. As expected, ALLN, MG132, and Bortezomib caused the accumulation of p21, indicating successful proteasome inhibition, while chloroquine and bafilomycin increased LC3-II levels, confirming lysosomal inhibition (Fig. 5C, E).

These results indicate that CXCR2 is mainly degraded through the proteasome pathway, consistent with reports of CXCR2 ubiquitination at Lys327 residue, a modification critical for its sorting and signaling [47].

To confirm Shp1 role in CXCR2 stability via the ubiquitin-proteasome pathway, we assessed CXCR2 ubiquitination in wild-type and Shp1-KO cells. 3XFlag-CXCR2 construct was overexpressed in both cell lines and similar transfection levels were obtained (Fig. 5F). Analysis of CXCR2 immunoprecipitated fractions, revealed a significantly stronger basal ubiquitination signal of CXCR2 in Shp1-KO cells (Fig. 5F, G), suggesting that loss of Shp1 promotes receptor ubiquitination, in lines with the lower levels of CXCR2 in these cells (Fig. 4D, E). This observation was also confirmed in the Shp1-KO cl.2, which displayed comparable alterations in CXCR2 modification and degradation (Fig. S1G–N).

Given these findings and the increased basal Ser347 phosphorylation in Shp1-KO cells, we hypothesized that this modification may drive CXCR2 ubiquitination. Thus, we generated a phospho-depleted mutant in which Ser347 was mutated to Alanine and then transfected into MCF7 cells to assess its ubiquitination (Fig. 5H). Interestingly, the typical ubiquitination ladder of the CXCR2 S347A mutant was significantly reduced compared to the wild-type CXCR2 receptor (Fig. 5H, I), providing compelling evidence that phosphorylation of Ser347 is important for CXCR2 ubiquitination.

The observed differences in receptor levels and degradation suggest an altered CXCR2-surface expression, potentially affecting IL8 response. To investigate this, we treated cells with IL8 for different time points (0–180 min) and used flow cytometry to assess membrane-associated CXCR2 with an antibody against its extracellular domain (Figs. 5J, K and S2F, G; see “Materials and methods”). At steady state, non-permeabilized Shp1-KO cells displayed ~20% lower surface CXCR2 compared to wild-type cells (Fig. 5J), consistent with the reduced total protein levels previously observed (Fig. 4D, E). In Fig. 5K, we present the quantification of flow cytometry experiments performed at the different time points shown in Fig. S2F, G, here summarized as a line graph. The graph shows that Shp1-KO cells underwent a faster loss of surface CXCR2. In contrast, wild-type cells exhibited an initial increase—likely reflecting ligand uptake [43]—followed by endocytosis and a return to baseline within 60 min. Shp1-KO cells lacked this early rise, with receptor levels instead declining steadily to baseline over ~3 h. These results suggest that Shp1 plays a role in stabilizing CXCR2 at the cell surface during IL8 stimulation. The initial transient increase in WT cells likely reflects receptor clustering or ligand-induced conformational changes that precede internalization, a regulatory step that appears to be impaired in the absence of Shp1. Instead, Shp1-KO cells show a progressive and accelerated loss of surface CXCR2, which may indicate enhanced receptor internalization and/or reduced recycling back to the plasma membrane. This altered trafficking could contribute to the overall lower steady-state levels of CXCR2 observed in Shp1-deficient cells and may ultimately dampen or dysregulate the cellular response to IL8.

**Fig. 6:**
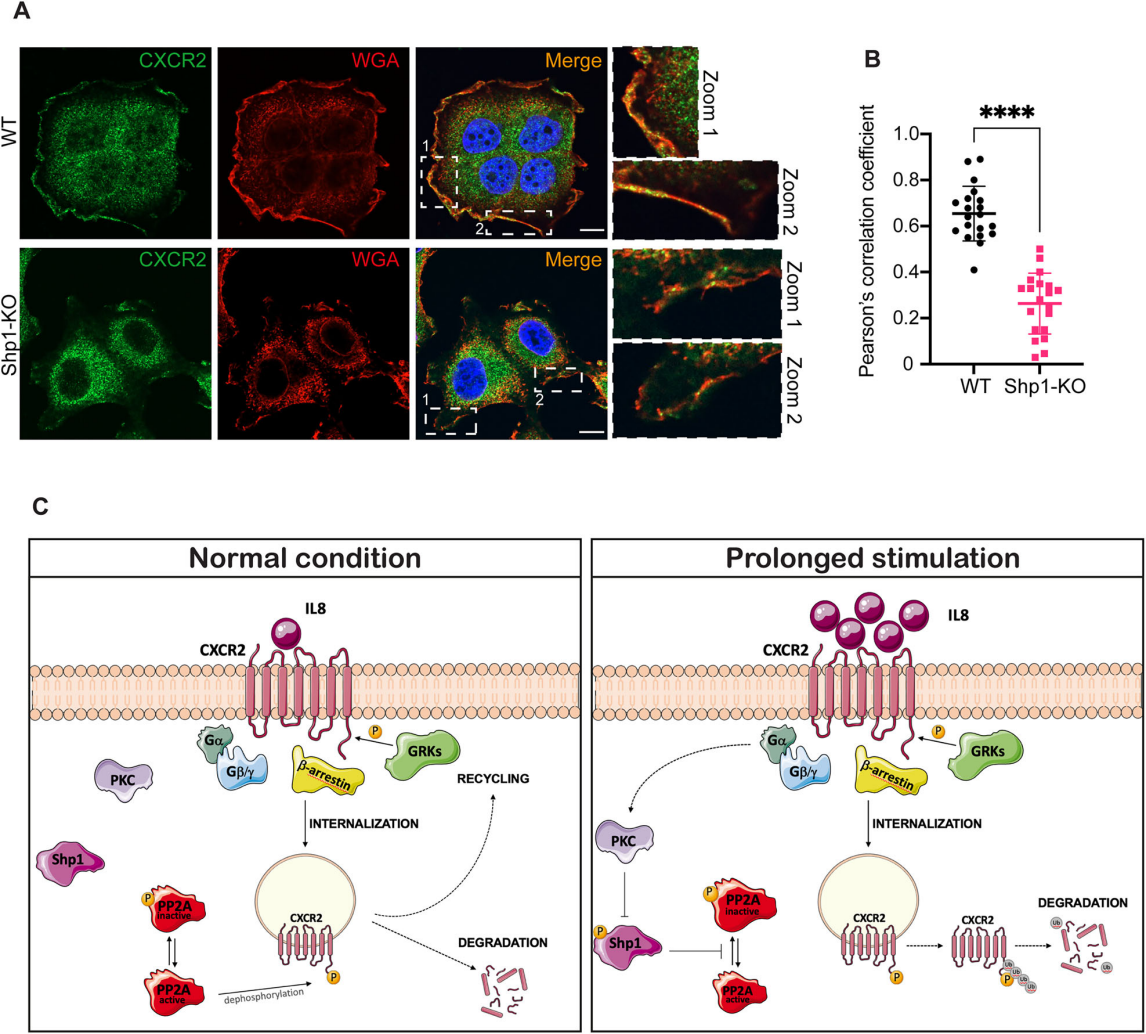
Increased plasma membrane localization of CXCR2 in MCF7 WT compared to MCF7 Shp1-KO cells. **A** Representative confocal microscopy images of MCF7 (WT) and MCF7 Shp1-KO cells (Shp1-KO). Cells were fixed and stained with Hoechst 33342 (nuclei, blue), WGA (plasma membrane, red), and an anti-CXCR2 antibody (endogenous CXCR2; green). Insets, right: Magnification of a plasma membrane section. Scale bars, 10 μm. **B** Pearson correlation coefficient of CXCR2 co-localization with WGA in (**A**). Data are means ± SD of three independent experiments. ****P < 0.0001 *versus* WT (Student’s t-tests). **C** Working model of CXCR2 trafficking and degradation under normal versus prolonged IL8 stimulation. Under normal stimulation (*left panel*), IL8 binding to CXCR2 activates downstream G-protein signaling (Gα/βγ). The receptor is subsequently phosphorylated by GPCR kinases (GRKs), leading to β-arrestin recruitment and receptor internalization. Internalized CXCR2 is either directed to recycling pathways back to the plasma membrane or targeted for degradation. Receptor fate is determined by the balance between phosphorylation and dephosphorylation, the latter mediated by active PP2A. Active PP2A promotes receptor recycling by removing phosphate groups from internalized CXCR2. Under prolonged stimulation (*right panel*), PKC-mediated inhibition of Shp1 phosphatase leads to inhibition of PP2A activity, preventing receptor dephosphorylation. This shift favors CXCR2 ubiquitination, leading to increased receptor degradation over recycling. This contributes to receptor desensitization and downregulation at the cell surface.

In support of this differential kinetic behavior, confocal microscopy analyses showed higher membrane localization of CXCR2 in wild-type compared to Shp1-KO cells (Fig. 6A, B).

Altogether, these data confirm that Shp1 is crucial for regulating CXCR2-surface expression dynamics, highlighting its role in receptor internalization kinetics and subsequent intracellular proteolytic degradation (Fig. 6C).

### Study of IL8/CXCR2/Shp1 pathway in different breast cancer subtypes

The experimental model used in this study, the MCF7-breast adenocarcinoma cell line, is classified as luminal A, which is ER/PR-positive and HER2-negative. Breast cancer is categorized into four main subtypes (luminal A, luminal B, HER2-positive, and triple-negative) based on receptor gene expression [48, 49].

To determine whether the IL8/Shp1 signaling observed in MCF7 cells also occurs in non-malignant cells, we analyzed MCF10A mammary epithelial cells. Under the same experimental conditions that induced Shp1 phosphorylation and invasion in tumor cells, IL8 failed to induce Shp1 phosphorylation in MCF10A, suggesting that IL8-mediated Shp1 activation is a tumor-specific event (Fig. S3A).

**Fig. 7:**
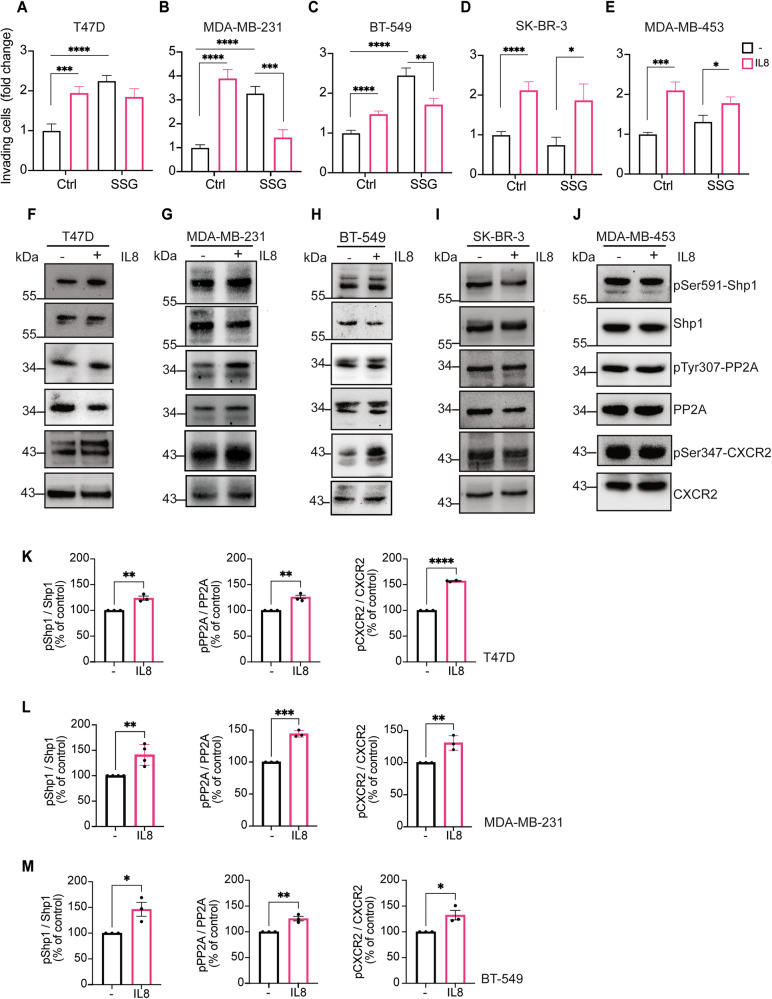
IL8 affects cell invasion and CXCR2-PP2A-Shp1 phosphorylation cascade in T47D, MDA-MB-231, BT-549, and not in SK-BR-3 and MDA-MB-453 breast cancer cells. Quantification of Matrigel invasion assays of T47D (**A**), MDA-MB-231 (**B**), BT-549 (**C**), SK-BR-3 (**D**), and MDA-MB-453 (**E**) cells after 24 h of treatment with PBS (vehicle control; −) or IL8 (250 ng/ml) in absence or presence of SSG (10 μM) (as indicated). Invasion was quantified by crystal violet staining, elution of invading cells, and data are presented as fold increase in invading cells. Data are mean ± SE of two independent experiments. ****P < 0.0001, ***P < 0.0005, **P < 0.005, *P < 0.05 versus Ctrl (vehicle control, PBS) (Student’s t-tests). Representative western blot using anti-phosphoserine 591 in Shp1 (pSer591-Shp1), anti-phosphotyrosine 307 in PP2A (pTyr307-PP2A) and anti-phosphoserine 347 in CXCR2 (pSer347-CXCR2) in T47D (**F**), MDA-MB-231 (**G**), BT-549 (**H**), SK-BR-3 (**I**) and MDA-MB-453 (**J**) cells treated with PBS (vehicle control; −) or IL8 (250 ng/ml; +) for 1 h. Total Shp1, PP2A, and CXCR2 were used as loading controls. Data are representative of at least three independent experiments. Molecular weight standards (kDa) are indicated on the left of each panel. **K**–**M** Densitometric quantification of the blots in (**F**–**H**). ****P < 0.0001, ***P < 0.0005, **P < 0.005, *P < 0.05 versus Ctrl (vehicle control, PBS) (Student’s t-tests).

To further strengthen our findings in luminal breast cancer models, we next included the ER-positive T47D cell line in our analysis (Fig. 7A). IL8 significantly increased cell invasion, while Shp1 inhibition by SSG abolished this effect and concomitantly enhanced basal invasion. These results confirm both the requirement of Shp1 for IL8-induced invasion and its intrinsic negative regulatory role in cell motility. Consistent with previous observations, IL8 triggered the Shp1-PP2A-CXCR2 phosphorylation cascade in T47D cells (Fig. 7F), and Shp1 inhibition resulted in an approximately 35% reduction in CXCR2 protein levels (Fig. S3B, C). Together, these findings demonstrate that the IL8/CXCR2/Shp1 signaling axis operates selectively in tumor cells and is conserved across luminal breast cancer models.

Given the novelty of the IL8/CXCR2/Shp1 pathway, we next extended our analysis to additional breast cancer subtypes with distinct receptor statuses and invasive behaviors, namely MDA-MB-231 and BT-549 (triple-negative) and SK-BR-3 and MDA-MB-453 (HER2-positive). Prior to these experiments, Shp1 expression was assessed in all the breast cancer cell lines by qRT-PCR and western blot, confirming the presence of the protein in each model (Fig. S3D, E).

IL8 promoted invasion in all models tested (Fig. 7B–E). Interestingly, SSG completely abolished IL8-induced invasion only in the triple-negative MDA-MB-231 and BT-549 cells, where it also enhanced basal invasiveness, confirming the inhibitory role of Shp1 in IL8-driven signaling (Fig. 7B, C). Although BT-549 cells are responsive to IL8 stimulation and sensitive to Shp1 inhibition, their response to IL8 was markedly weaker compared to all other cell models. Whether this limited responsiveness is related to the low levels of Shp1 expression detected in BT-549 cells (Fig. S3D, E) remains to be determined. In contrast, in SK-BR-3 and MDA-MB-453 cells, inhibition of Shp1 had no effect on either basal or IL8-induced invasion, while IL8 stimulation alone promoted invasion similarly to other cell lines (Fig. 7D, E).

As these results indicate subtype-specific signaling, we further investigated whether IL8 could activate the Shp1-PP2A-CXCR2 cascade identified in MCF7 cells (Fig. 4A). Intriguingly, IL8 treatment increased the phosphorylation of PP2A and CXCR2, together with Shp1 phosphorylation, only in MDA-MB-231 and BT-549 cells, but not in SK-BR-3 or MDA-MB-453 cells (Fig. 7G–J).

Collectively, these results validate the inhibitory role of Shp1 in IL8-induced pro-invasive signaling and suggest that the IL8/CXCR2/Shp1 axis is functionally active in luminal and triple-negative breast cancers, but not in HER2-positive subtypes.

### Shp1 expression and its relationship with CXCR2 and IL8 in breast cancer subtypes

**Fig. 8:**
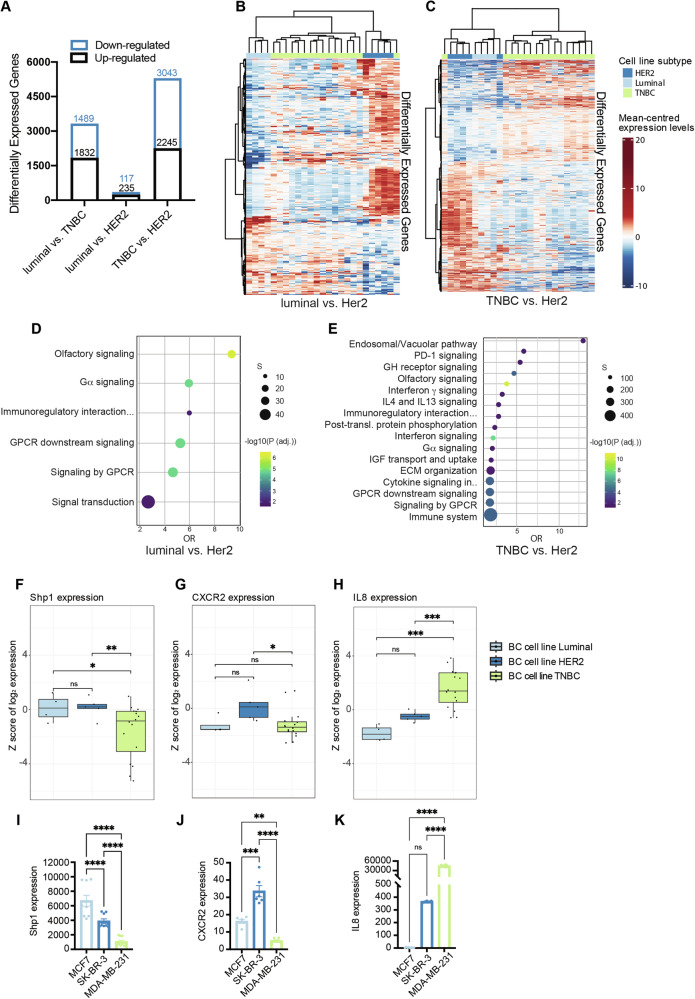
Analysis of gene expression signatures in luminal, Her2 and TNBC cell types. **A** The plot shows the number of the differentially expressed genes (DEG) identified between the contrasts “luminal vs TNBC,” “luminal vs HER2” cell lines, and “TNBC vs HER2” cell lines defined using adjusted *p* value < 0.05 and fold change ≥2. Note that the numbers on Y axis represent the total number of selected genes found for each contrast. The genes downregulated and upregulated are shown in light blue and black, respectively. Heatmap of significant genes for comparison “luminal vs HER2” cell lines (**B**) and “TNBC vs HER2” cell lines (**C**). The Heatmap shows gene intensity per sample relative to the average level across all samples. Individual genes are shown on the Y axis, while samples are shown along the X axis. Red and blue cells correspond to higher and lower RNA-seq levels, respectively. A maximum of 1000 features and 1000 samples are shown (selected at random when the number exceeds these limits). **D**, **E** Dot plot of significantly enriched manually curated Reactome pathways of genes identified in (**B**, **C**). Enrichment results were plotted as manually curated Reactome pathways (Y axis) against odds ratio (OR, X axis), calculated as the ratio of significant genes observed over that which could be expected by chance. Points are colored by −log10(adjusted *P* value) value and sized by the number of member genes within each manually curated Reactome pathway. Boxplots of expression of Shp1 (**F**), CXCR2 (**G**), and IL8 (**H**) across all samples grouped by breast cancer cell lines. Each dot represents a sample and expression (Y axis) has been summarized by tissue type (X axis). Expression is presented as Z-scores of the TMM normalized values. ***P < 0.005, **P < 0.005; *P < 0.05 (One-way analysis). Analysis by qRT-PCR of mRNA levels of Shp1 (**I**), CXCR2 (**J**), and IL8 (**K**) in MCF7, SK-BR-3, and MDA-MB-231 cells. GAPDH was used as housekeeping gene. Data are means ± SE of three independent experiments performed in triplicate. ****P < 0.0001, ***P < 0.0005, **P < 0.005 (One-way analysis).

To gain insight into the molecular characteristics underlying the divergent cellular responses observed across different breast cancer subtypes, we performed a comprehensive analysis of transcriptomic data from human breast cancer cell lines available in the GEO repository. We selected the GSE58135 dataset, which profiles gene expression across 25 breast cancer cell lines encompassing all major molecular subtypes, with multiple independent models for each group (luminal, HER2-positive, and triple-negative; see list in Supplementary Table S1). This provides a robust and representative basis for comparative analyses across biologically distinct subtypes. After applying thresholds of adjusted *p* value < 0.05 and fold change ≥2, we identified 3321 differentially expressed genes (DEGs) between luminal and TNBC cell lines (1832 upregulated and 1489 downregulated), 352 DEGs between luminal and HER2 cell lines (235 upregulated and 117 downregulated), and 5288 DEGs between TNBC and HER2 cell lines (2245 upregulated and 3043 downregulated) (Fig. 8A–C). Volcano plots of the DEGs are shown in Fig. S3F, G.

Based on the identified DEGs and the observation that the IL8/Shp1 pathway regulates invasive potential in luminal and TNBC cell lines, but not in HER2 cell lines, we conducted a Reactome pathway analysis of DEGs by comparing responsive and unresponsive cells, specifically “luminal vs. HER2 cell lines” and “TNBC vs. HER2 cell lines” (Fig. 8D, E). The Reactome database provides extensive annotations for a wide range of molecular and cellular processes, such as signaling, transport, cell motility, metabolism, immune function, and more. A total of 6 and 17 manually curated Reactome pathways were found to be significantly enriched (adjusted *P* value < 0.05 and ≥2 features per set) in the comparison “luminal vs. HER2 cell lines” and “TNBC vs. HER2 cell lines,” respectively. Notably, shared enriched pathways included the endosomal/vacuolar transport system, PD-1 signaling, cytokine-mediated pathways, and G-alpha subunit-mediated signaling. These findings reinforce the central involvement of GPCR-related signaling in subtype-specific regulatory mechanisms. Within the GSE58135 dataset, we also examined the expression of *PTPN6* (encoding Shp1), *CXCR2*, and *IL8* across breast cancer subtypes. Shp1 was significantly reduced in TNBC cell lines compared to both HER2-positive and luminal cell lines (Fig. 8F). CXCR2 expression did not significantly differ between TNBC and luminal or HER2 and luminal subtypes, but it was significantly downregulated in TNBC compared to HER2 cell lines (Fig. 8G). IL8 was markedly higher in TNBC cells compared to both HER2 and luminal cell lines, with no significant difference between the latter two subtypes (Fig. 8H).

To experimentally validate these transcriptomic observations, we performed qRT-PCR analysis in both MCF7 (luminal A), SK-BR-3 (HER2-positive), and MDA-MB-231 (TNBC) cell lines. The expression trends for *Shp1*, *CXCR2*, and *IL8* in these lines aligned closely with the RNA-seq data, confirming lower Shp1 and CXCR2 and higher IL8 expression in TNBC cells (Fig. 8I–K).

In summary, these data confirm that specific gene signatures are linked to different breast cancer subtypes. They also highlight the role of GPCRs and their signaling pathways in these differences, indicating the Shp1-mediated regulation of CXCR2 is a crucial component of this process and, accordingly, a promising therapeutic target.

## Discussion

The tyrosine phosphatase Shp1 acts as a tumor suppressor in various cancers, including breast cancer, where it inhibits cell proliferation and invasion by dephosphorylating receptors like EGFR and HER2 [6, 7]. In this study, we explored a different aspect of Shp1 action and now report its involvement in the IL8-CXCR2 activation and the resulting protumor activity; this provides new insights into Shp1 signaling and breast cancer progression.

The IL8-CXCR2 axis plays dual roles in cancer, both activating the immune response and shaping the EMT to favor tumor progression [23], via promoting proliferation and metastasis through autocrine signaling, activation of tumor-promoting pathways, EMT, and chemotherapy resistance [24]. IL8 binds to CXCR2, triggering downstream signaling, including PKC activation with the phosphorylation of substrates such as Shp1 [40–42]. We show that IL8 phosphorylates Shp1 on Ser591, reducing its activity and thereby preventing the dephosphorylation of PP2A at the inhibitory Tyr307 site. This results in PP2A inactivation, which prevents catalysis. Based on these findings, we propose that IL8-mediated Shp1 phosphorylation inhibits Shp1 function, blocking PP2A and consequently sustaining CXCR2 phosphorylation, with the result of limiting cancer cells response to IL8 (Fig. 6C).

CXCR2 phosphorylation is crucial for modulating ligand responsiveness [46, 50], maintained through a constitutive phosphorylation-dephosphorylation cycle regulated by GRKs and PP2A [43]. Agonist-induced phosphorylation facilitates CXCR2 internalization into early endosomes, where dephosphorylation enables recycling and restores cellular responsiveness [51, 52]. We have identified the mechanism by which IL8-induced CXCR2 hyperphosphorylation attenuates responsiveness by enhancing receptor ubiquitination and degradation. Prolonged IL8 exposure maintains CXCR2 in a phosphorylated state, leading to enhanced receptor degradation. Notably, this process is initiated by IL8-driven Shp1 phosphorylation, which acts as a molecular switch. Accordingly, in the absence of Shp1, IL8 fails to induce CXCR2 phosphorylation, emphasizing the regulation that Shp1 imposes on this process.

Another novel aspect of this study is the regulatory mechanism controlling CXCR2 stability via the ubiquitin-proteasome system, modulated by Ser347 phosphorylation. In Shp1-KO cells, elevated CXCR2 phosphorylation increases polyubiquitination and degradation, without altering CXCR2 mRNA levels. Proteasome inhibition or Shp1 re-expression restores CXCR2 levels, reinforcing the role of Shp1 in stabilizing CXCR2 by modulating its phosphorylation state and subsequent ubiquitination. Together, these findings define Shp1 as a master regulator of CXCR2 signaling and IL8 responsiveness.

These results also point to a link between phosphorylation and ubiquitination, further supported by the observation that the phospho-deficient CXCR2 S347A-mutant exhibits reduced ubiquitination. The crosstalk between these post-translational modifications is a concept well-documented in various signaling proteins where phosphorylation-dependent ubiquitination controls protein stability, trafficking, and degradation [53, 54]. How phosphorylation of Ser347 regulates the ubiquitination of CXCR2 remains elusive. Previous data identified the conserved Lys327 residue on CXCR2 as a key ubiquitin acceptor upon IL8 stimulation [47]. Given the spatial proximity between Lys327 and Ser347, we speculate that phosphorylation serves as a priming signal for ubiquitination, either creating an E3 ligase recognition site or inducing conformational changes that expose Lys327. This model is consistent with evidence from other GPCRs. For example, phosphorylation of Ser324/325 in CXCR4 promotes binding to the E3 ligase AIP4, and mutation of these sites reduces receptor ubiquitination and degradation [55]. Similarly, phosphorylation of serine/threonine residues in the C-terminal tail of CXCR7 (Ser350, Thr352, and Ser355) has been shown to regulate receptor internalization and ligand degradation [56]. These parallels reinforce the concept that phosphorylation of C-terminal serine residues is a key determinant of GPCR ubiquitination and turnover. Nonetheless, the specific E3 ligase targeting CXCR2 remains unidentified, and additional phosphorylation sites beyond Ser347 may also contribute to its regulation.

Beyond regulating CXCR2 expression and IL8 signaling, Shp1 also modulates breast cancer cell migration and invasion. Shp1 inhibition blocks IL8-induced migration and reduces basal invasiveness, consistent with studies showing that Shp1 loss enhances activation of motility-promoting pathways like EGFR, HER2, and cortactin signaling [4, 6, 7]. Interestingly, in the absence of functional Shp1, IL8 inhibits rather than promotes invasiveness, implying that IL8 effects cannot be solely attributed to Shp1.

Our results show that Shp1-mediated IL8-induced migration is cell line-dependent. In luminal breast cancer models, including MCF7 and T47D cells, IL8 stimulation promoted invasion through activation of the Shp1–PP2A–CXCR2 cascade, while Shp1 inhibition enhanced basal invasiveness and abolished the IL8 response. A similar behavior was observed in triple-negative models (MDA-MB-231 and BT-549), confirming the negative regulatory role of Shp1 in this context. In contrast, in HER2-positive cell lines (SK-BR-3 and MDA-MB-453), inhibition of Shp1 had no significant impact on either basal or IL8-induced invasion, while IL8 stimulation remained effective in promoting invasiveness. Given the reported interaction of Shp1 with the HER2 receptor [7], this lack of effect was unexpected. A plausible explanation is that active HER2 sequesters Shp1, limiting its availability to engage with other signaling pathways, whereas in HER2-negative cells, Shp1 remains free to modulate alternative receptors such as CXCR2.

This subtype-dependent behavior prompted us to further explore the molecular determinants underlying these differences. Transcriptomic analyses comparing responsive (luminal, TNBC) and unresponsive (HER2-positive) cells revealed a strong association between cytokine-mediated and G-alpha signaling pathways; these findings underscore the involvement of GPCR-downstream signaling in Shp1-mediated regulation and the role of Shp1 in the regulation of other signals beyond the well-known HER2 pathway. Analysis of Shp1 expression across breast cancer subtypes indicated that low levels of Shp1 (downregulated in TNBC alongside with reduced CXCR2 and elevated IL8) are associated to the most aggressive disease form. Furthermore, the correlation between Shp1 transcripts and poorer survival probability in breast cancer patients (Fig. S1C) becomes even more pronounced when the analysis is restricted to a cohort of TNBC patients, emphasizing the prognostic significance of Shp1 in this aggressive breast cancer subtype.

Although in vitro models have limitations, our findings indicate that Shp1 role in cancer invasion is subtype-specific, mainly affecting luminal and TNBC cells. Given the limited targeted therapies for TNBC, this previously unknown role of Shp1 in modulating IL8 and CXCR2 signaling becomes crucial and offers a promising strategy to fight cancer invasion and metastasis. Shp1, already targeted in anticancer strategies for its inhibition of STAT3 phosphorylation [16], can be modulated by natural compounds, small molecules, and FDA-approved drugs like sorafenib and regorafenib [9, 57]. Our data suggest these drugs may exert antitumor effects not only through the inhibition of STAT3 signaling but also by modulating the Shp1-CXCR2 axis. Thus, agents that upregulate or activate Shp1 could provide broader antitumor benefits by targeting multiple oncogenic pathways.

Beyond breast cancer, our results may hold broader oncological significance. The IL8-CXCR2 axis is known to play a role in the progression of other malignancies, including lung, pancreatic, and prostate cancers, as well as melanoma [23]. It is therefore plausible that Shp1 may act as a regulator of this pathway in additional tumor types, extending the relevance of our results beyond the breast cancer setting. Moreover, the identification of Shp1 as a regulator of CXCR2 stability opens the way to therapeutic strategies based on combinatorial approaches. Agents that enhance Shp1 activity or prevent its inhibitory phosphorylation could be tested in combination with CXCR2 antagonists, immune checkpoint inhibitors, or conventional chemotherapies, potentially enhancing efficacy and overcoming resistance. These possibilities underline the translational potential of our work and call for further studies in different oncological contexts.

In summary, our findings position Shp1 as a central node in the regulation of IL8-CXCR2 signaling through coordinated control of phosphorylation and ubiquitination. This dual regulatory role establishes Shp1 as both a biomarker and a potential therapeutic target in aggressive breast cancers, particularly TNBC.

## Materials and methods

### Antibodies, reagents, and constructs

The antibodies used were: polyclonal anti-CXCR2 (PA5-102662), polyclonal anti-pSer347-CXCR2 (PA5-104850), PerCP-conjugated anti-CXCR2 (46-1829-42), monoclonal anti-PP2Aα (MA5-18060), polyclonal anti-Tyr307-PP2Aα (PA5-36874) and Alexa Fluor 555-conjugated WGA (W32464) from Invitrogen (Thermo Fisher Scientific, Waltham, MA, USA), monoclonal anti-Shp1 (26516), polyclonal anti-Vinculin (4650), monoclonal anti-GAPDH (97166) monoclonal anti-LC3 (12741), monoclonal anti-Ubiquitin (20326), monoclonal anti-p21 (2947), anti-rabbit IgG/HRP-linked secondary antibody (7074) and anti-mouse IgG/HRP-linked secondary antibody (7076) from Cell Signaling Technology (Danvers, MA, USA), polyclonal anti-pSer591-Shp1 (Ab41436, Abcam, Cambridge, UK), monoclonal anti-β-Tubulin (E-AB-20033, Elabscience, Houston TX, USA).

Reagents included human IL8 (130-122-360, Miltenyi Biotec, Bergisch Gladbach, Germany), SSG inhibitor Sodium Stibogluconate (567565), Chloroquine diphosphate salt (C6628), PKCi Bisindolylmaleimide I (203290) and Cycloheximide (01810) from Merck Millipore (Burlington, MA, USA), MG132 (2194) and Bafilomycin A1 (54645) from Cell Signaling Technology, ALLN (208719, Calbiochem, EMD Chemicals, San Diego, CA, USA), Bortezomib (sc-217785, Santa Cruz Biotechnology, Dallas, TX, USA).

Expression vectors used were Shp1 and Shp1-C455S (kindly provided by F.D. Böhmer, CMB, Friedrich Schiller University of Jena, Germany) and 3xFlag-CXCR2 (EX-A0079-M12, GeneCopoeia, Rockville, MD, USA).

### Cell culture and treatments

MDA-MB-231, MCF10A, T47D, and SK-BR-3 cell lines were from the American Type Culture Collection (ATCC, Manassas, VA, USA). MCF7 and MCF7 Shp1-KO were kindly provided by Dr. Maddika (Centre for DNA Fingerprinting & Diagnostics, India). The knockout was validated by assessing Shp1 expression levels through western blot (Fig. 1B). The generation of these cell lines was previously described in [58]. MDA-MB-453 and BT-549 were kindly provided by Dr. Laura Cerchia (Institute of Endotypes in Oncology, Metabolism and Immunology “Gaetano Salvatore”, National Research Council). MCF7, MCF7 Shp1-KO and MDA-MB-231 were cultured in DMEM; MCF10A were cultured in DMEM/F12 supplemented with Horse Serum (5%), EGF (20 ng/mL), Hydrocortisone (0,5 µg/mL), Cholera Toxin (100 ng/mL) and Insulin (10 µg/mL); SK-BR-3, T47D, MDA-MB-453 and BT-549 in RPMI-1640, all supplemented with 10% FBS and 1% L-glutamine (GIBCO, Thermo Fisher Scientific), at 37 °C and 5% CO_2_. Experiments were conducted up to 10 passages, and all cells were routinely confirmed to be mycoplasma negative.

Transient transfections were performed with Lipofectamine LTX (15338, Invitrogen, Thermo Fisher Scientific) according to the manufacturer’s instructions and analyzed 24–48 h later.

For RNA interference, cells were transfected with 200 nM of the siGENOME™ SMARTpool® reagents (Dharmacon, Lafayette, CA, USA) containing four pooled siRNA duplexes against human Shp1 using Lipofectamine RNAiMAX (Invitrogen, Thermo Fisher Scientific) according to the manufacturer’s instructions.

For IL8 treatments, cells (3 × 10⁵/well, 6-well plate) were serum-starved (1% FBS) for 16 h, then treated with 250 ng/mL IL8 with/without inhibitors, as indicated in the text and/or Figure legends. For PKCi experiments, cells were pretreated with 1 μM PKCi for 30 min before IL8 stimulation.

### Wound healing and invasion assays

An Ibidi Culture Insert (Ibidi GmbH, Gräfelfing, Germany) was used to seed 3 × 10⁴ MCF7 wild-type and Shp1-KO cells. After overnight incubation, the insert was removed to create a 500 µm gap. Wound closure was monitored over 24 h using ZOE Fluorescence Cell Imager (Bio-Rad, Hercules, CA, USA) and analyzed with ImageJ Fiji software. The wound width was calculated by measuring the area between the edges of the wound at time T0 (time of wounding) and T24 h (24 h after wounding).

Invasion assays were performed using transwell chamber (3422, Corning, NY, USA) coated with 1.5 mg/mL Matrigel (354234, Corning, NY, USA). The cells (8 × 10^4^/well) were treated as indicated and then seeded to the upper part of the chamber in serum-free medium. In the lower chamber, medium containing 1% FBS in absence or presence of 250 ng/mL IL8 served as chemoattractant. After 16 h, invaded cells were fixed in 4% paraformaldehyde solution and stained with Crystal Violet (0,5% in 25% MetOH) for 30 min. Cell staining was eluted with 33% acetic acid, transferred to a 96-well plate, and absorbance measured at 590 nm. For quantitative analysis, cells were plated at different concentrations and stained using the same protocol. Absorbance at 590 nm was plotted against concentration to create a standard curve.

### Immunofluorescence analysis

Cells (5 × 10^4^) were grown on coverslips, fixed in 4% paraformaldehyde for 10 min, permeabilized with blocking solution (0.05% saponin, 0.5% BSA, 50 mM NH4Cl) for 20 min, washed three times with PBS, and incubated with Alexa Fluor™ 488-conjugated phalloidin (A12379, Invitrogen, Thermo Fisher Scientific) for 1 h at room temperature. Coverslips were mounted with Mowiol. Morphological analysis was performed as described in [39].

For CXCR2 staining, WT and Shp1-KO MCF7 cells were incubated at 4 °C (on ice) for 60 min to reduce membrane trafficking and endocytosis, thereby enhancing receptor staining at the plasma membrane. Following incubation, cells were fixed and stained with 5 μg/ml Alexa Fluor 555-conjugated wheat germ agglutinin (WGA), commonly used to label plasma membrane glycoproteins, and with a polyclonal anti-CXCR2 antibody, incubated overnight at 4 °C. Cells were then washed three times with PBS, and coverslips mounted with Mowiol. Image acquisition was performed using a Zeiss LSM 980 confocal microscope equipped with an Airyscan module (Carl Zeiss, Göttingen, Germany). Co-localization between WGA and CXCR2 was assessed by calculating the Pearson correlation coefficient using the JACoP plugin in Fiji [59]. Data analysis was performed using GraphPad Prism (version 10, GraphPad Software, La Jolla, CA, USA).

### Western blot and immunoprecipitation

Cells were washed twice with ice-cold PBS and lysed on ice in buffer containing 20 mM Tris-HCl, pH 8.0, 150 mM NaCl, 1% Triton-X100, 5 mM Na_3_VO_4_, 30 mM β-glycerophosphate, and 10 mM NaF, supplemented with the EDTA-free protease inhibitor cocktail (58698000, Roche, Basel, Switzerland). The lysates were homogenized by three passages through a 26-gauge needle and 10 min on wheel at 4 °C, centrifuged at 13,000 rpm for 10 min, and the supernatant was collected. Protein concentration was determined by Bradford assay, proteins analyzed by SDS-PAGE, and filters were developed by using the ChemiDoc MP Imaging System. Western blotting was performed with the indicated antibodies.

For Shp1 immunoprecipitation, 1 mg of lysate was incubated 1:100 with anti-Shp1 antibody (overnight, 4 °C, shaking). Then 50 μl protein A Sepharose beads (17127901, Cytiva, Marlborough, MA, USA) were added for a further 1 h incubation (4 °C, shaking). For 3XFLAG-CXCR2 immunoprecipitation, 30 μl anti-FLAG M2 agarose beads (A2220, Sigma-Aldrich, St. Louis, MO, USA) were added to 1 mg of cell lysates (overnight, 4 °C, shaking). The day after, the beads were washed with lysis buffer, and denatured complexes were eluted with SDS-sample buffer.

### In vitro phosphatase assay

Shp1 was immunoprecipitated from MCF7 cells as described above. The immunoprecipitated protein was washed three times with lysis buffer, three times with lysis buffer without phosphatases inhibitors, and twice with phosphatase buffer (100 mM Na-Hepes, pH 7.4, 150 mM NaCl, 1 mM EDTA, and 10 mM DTT). The immunoprecipitated was incubated with 2 mM p-Nitrophenyl phosphate (pNPP; 128860100, Acros Organics, Geel, Belgium) for 1 h at 37 °C. The reactions were quenched with 100 μl 10 N NaOH, and the enzymatic activity was calculated from the amount of the released p-nitrophenolate, determined from its spectrophotometric absorbance at 405 nm. Control immunoprecipitated samples were analyzed by SDS-PAGE, and the amount of immunoprecipitated Shp1 was determined by western blotting.

### qRT-PCR

RNA was extracted by using the RNeasy Mini Kit (74106, Qiagen, Hilden, Germany), and 1 µg was reverse transcribed following the QuantiTect Reverse Transcription Kit (205313, Qiagen). 50 ng of cDNA were amplified by using the Syber Green Master Mix (LightCycler 480 SYBR Green I Master, 04887352001, Roche) and specific primers for Shp1 (forward 5′-CCAGGATGGTGAGGTGGTTTC-3′; reverse 5′-AAGTCACCCTGGTTCTTGCG-3′), CXCR2 (forward 5′-TTCCGAAGGACCGTCTACTCA-3′; reverse 5′-AGGGTGAATCCGTAGCAGAAC-3′), IL8 (forward 5′-GAACTGAGAGTGATTGAGAGTGG-3′; reverse 5′-TCTGCACCCAGTTTTCCTTG-3′), GAPDH (forward 5′-CCACATCGCTCAGACACCAT-3′; reverse 5′-AGTTAAAAGCAGCCCTGGTGAC-3′), actin (forward 5′-GCACTCTTCCAGCCTTCC-3′; reverse 5′-TGTCCACGTCACACTTCATG-3′). Light Cycler 480 II thermocycler (Roche) was used for the reaction and CT data analysis. Expression was normalized to housekeeping genes (actin or GAPDH) using the 2^−ΔCT^ method.

### CXCR2 degradation studies

MCF7 and MCF7 Shp1-KO cells (3 × 10⁵/well, 6-well plate) were seeded and treated the following day. For Cycloheximide chase assay, cells were pretreated with 25 µg/mL CHX 1 h before IL8 stimulation. For ubiquitin-proteasome inhibition, MCF7 Shp1-KO cells were treated with 20 µM ALLN, 25 µM MG132, or 1 µM Bortezomib for 4 h. For lysosomal inhibition, cells were treated with 100–300 nM Bafilomycin A1 or 50–100 µM Chloroquine for 4 h. Cells were then processed and CXCR2 levels analyzed by western blotting.

### Ubiquitination assay

Cells were transfected with 3XFlag-CXCR2, and Bortezomib was added after 24 h (for additional 16 h in 1%FBS-containing medium). Lysates were precipitated using anti-FLAG M2 agarose beads (as described above), followed by western blotting analysis with anti-ubiquitin antibody.

### Flow cytometry analysis

Cells were trypsinized, washed with PBS, and 5 × 10^5^ cells for each condition were labeled with 125 ng of PerCP-conjugated anti-CXCR2 antibody (30 min on ice, protected from light). Cells were washed, resuspended in 400 μL of FACS buffer (0,1% FBS in PBS), and CXCR2 expression was assessed using the Becton Dickinson (BD) FACS AriaIII instrument. The mean fluorescent intensity of analyzed cells was used to quantify CXCR2-surface expression, with data presented as a percentage relative to the initial surface receptor levels in untreated cells.

### RNA-seq analysis

FASTQ files containing bulk RNA-seq expression data pertaining to 168 breast tissue samples were accessed from the European Nucleotide Archive (project PRJNA251383). Of these 168 samples, 26 were derived from breast cancer cell lines encompassing three molecular subtypes: 17 triple- negative, 5 HER2+, and 4 luminal. These data were generated as part of a previously published study [60]. There were between 27.9 and 161.8 million paired-end reads per sample. Reads were mapped to the reference human transcriptome (GRCh38, GENCODE v44) using Salmon v1.9.0. Mapped reads ranged from 17 to 117 million reads per sample. A read count filter was applied using the Bioconductor edgeR package to exclude features with low expression. Read counts for 39,597 features were retained for downstream analysis. The raw counts data were subjected to quality control (QC) and exploratory data analysis (EDA) consisting of application of four automatic outlier detection tests (sum of Euclidean distance to other samples, Kolmogorov-Smirnov test statistic, mean Pearson correlation with other samples, and Hoeffding’s D statistic), principal component analysis, and inter-sample correlation analysis. For QC and EDA purposes, normalization of the raw counts data was performed using the VST method. Two samples, including a TNBC cell line sample (SRR1313083), were identified as outliers during this process and excluded from further analysis. Normalization of the raw counts data for the remaining samples was performed using the trimmed mean of M-values method [61], followed by voom transformation [62]. These normalized data were used for differential expression analysis. Differential expression analysis was performed using Bioconductor limma package, and pairwise contrasts were performed between the molecular subtypes. Genes were considered differentially expressed if they met a false discovery rate (FDR) adjusted *p* value threshold of <0.05 and fold change ≥2. Functional enrichment analysis was performed by conducting over-representation analysis using the Bioconductor cluster Profiler package, based on the Reactome database. Reactome pathways terms were considered over- represented if they met a false discovery rate (FDR) adjusted *p* value threshold of <0.05 and included at least two differentially expressed genes. Data analysis and visualizations, including heatmaps and volcano plots, were generated using R v4.3.2 and the following packages: clusterProfiler v4.10.1, ComplexHeatmap v2.18.0, DESeq2 v1.42.1, edgeR v4.0.16, ggplot2 v3.5.0, GO.db v3.18.0, limma v3.58.1, org.Hs.eg.db v3.18.0, plotly v4.10.4, and tximport v1.30.0.

### Databases

Gene expression-survival correlations were analyzed with GEPIA2 (http://gepia2.cancer-pku.cn) and Kaplan–Meier plotter (https://www.kmplot.com/analysis/).

### Statistics

Data were analyzed using Prism 9.0 (GraphPad Software, La Jolla, CA, USA). Error bars represent standard error or standard deviation as indicated in the text. Statistical significance was determined by paired t-tests or one-way ANOVA. *P* values are indicated as: ****P < 0.0001, ***P < 0.0005, **P < 0.005, *P < 0.05.

## Supplementary information

Supplementary Information Files

Original Western Blots

## Data Availability

Data will be provided by the corresponding authors upon request.
