# Supplementary material for: Shp1 phosphatase regulates CXCR2 protein stability and IL8-mediated invasiveness in breast cancer
Source: Cell Death Dis. 2026 Mar 2;17(1):297. doi: 10.1038/s41419-026-08516-4 (PMC13039404; doi:10.1038/s41419-026-08516-4)
Supplement: Supplementary file 1 — Supplementary Information Files [file 41419_2026_8516_MOESM1_ESM.docx]

**SUPPLEMENTARY INFORMATION FILES**

The Supplementary Information Files include Supplementary Figures 1,2 and 3, their corresponding figure legends, the Supplementary Table 1 and the original western blots.

**Supplementary Fig. 1 Characterization of Shp1-KO clone 2 and analysis of CXCR2 signaling and post-translational regulation.**

*A.* Representative images of wound-closure assays of MCF7 (WT) and MCF7 Shp1-KO clone 2 (Shp1-KO cl.2) cells. *B.* Representative images of wound closure assays of Shp1-KO cells clone 2 (Shp1-KO cl.2) after 24 h of treatment with PBS (vehicle control; -) or IL8 (250 ng/ml). *C and D.* Quantification of the extent of wound closure calculated by analyzing the scratched area covered by cells in A and B (respectively) after 24 h using ImageJ software. *E and F.* Quantification of Matrigel invasion assays of MCF7 cells treated as in A and B. Invasion was quantified by crystal violet staining elution of invading cells and data are presented as invading cells (expressed as fold increase in invading cells). Data are mean ± SD of three independent experiments performed in triplicate. ****P< 0.0001, *P< 0.05 versus WT cells or vehicle control (Student’s t-tests). *G.* Representative western blot using anti-Shp1and anti-CXCR2 specific antibodies in MCF7 WT or MCF7 Shp1-KO clone 2 (Shp1-KO cl.2) cells. GAPDH was used as loading control. Data are representative of three independent experiments. Molecular weight standards (kDa) are indicated on the left of each panel. *H*. Representative western blot using anti-phosphotyrosine 307 in PP2A (pTyr307-PP2A) and anti-phosphoserine 347 in CXCR2 (pSer347-CXCR2) specific antibodies in MCF7 Shp1-KO cl.2 cells treated with PBS (vehicle control; -) or IL8 (250 ng/ml; +) for 1 h. Total CXCR2, PP2A and β-tubulin were used as loading controls. Data are representative of at least three independent experiments. Molecular weight standards (kDa) are indicated on the left of each panel. *I.* Representative western blot using anti-CXCR2 specific antibody in MCF7 Shp1-KO cl.2 cells treated with DMSO (vehicle control; Ctrl) or 1 μM Bortezomib for 4 hours. GAPDH was used as loading control. Data are representative of two independent experiments. Molecular weight standards (kDa) are indicated on the left of each panel. *J.* Densitometric quantification of the blot in I. *K.* Representative western blot using anti-ubiquitin antibody in the precipitate CXCR2 fraction (IP) from MCF7 WT transfected with an empty 3xFlag-vector or with 3xFlag-CXCR2 and from MCF7 Shp1-KO cl.2 cells transfected with 3xFlag-CXCR2. The cells were treated with Bortezomib (1 μM) for 16 hours and then the lysates were immunoprecipitated using anti-Flag beads (see Methods). Ubiquitin staining of 3xFlag-CXCR2 is shown with two different exposure times (low and high) for better interpretation. The protein levels of total 3xFlag-CXCR2 were detected with anti-Flag antibody and indicate comparable amounts of transfected protein (lower panel). Data are representative of two independent experiments. Molecular weight standards (kDa) are indicated on the left of each panel. *L.* Quantification of ubiquitinylated receptor as in K.

**Supplementary Fig. 2** **Shp1 expression correlates with patient survival, decreased tumor invasiveness, and CXCR2 surface expression in breast cancer cells.**

*A-C.* Association of Shp1 expression with patient survival and differential gene expression profiles in breast cancer cell lines. *A.* TCGA based validation and survival analysis on GEPIA of Shp1 expression in breast cancer patients over a period of 250 months. The red line represents high Shp1 expression, while the blue line represents low Shp1 expression. *B and C.* Kaplan-Meier survival curve analyzing the correlation between Shp1 levels and percentage of survival in a total cohort of breast cancer patients (B) and in the specific subgroup of TNBC patients (C) over a period of 80 months. The red line represents high Shp1 expression, while the black line represents low Shp1 expression.

*D.* Invasion assay performed in MCF7 cells transfected with either control (non-targeting) or Shp1-specific siRNAs for 24 h and then transferred to the upper side of a Matrigel-coated transwell chamber, in the presence or absence of IL8 (250 ng/mL, 16 h). Invasion was quantified by crystal violet staining and elution of invading cells. Data are presented as fold increase in invading cells. Values represent the mean ± SD of two independent experiments performed in duplicate. ****P < 0.0001 versus non-targeting siRNA control (Student’s t-test). *E.* Western blot confirming efficient Shp1 knockdown and showing CXCR2 protein levels in the same experimental conditions. GAPDH was used as a loading control. Data are representative of two independent experiments. Molecular weight standards (kDa) are indicated on the left of each panel. *F and G.* Flow cytometry analysis of CXCR2 surface expression in response to IL8. Cytofluorimetric histograms of MCF7 (F) and MCF7 Shp1-KO (G) cells treated with IL8 (250 ng/ml) for the indicated time points, stained with PerCP-Cy5.5-conjugated anti-CXCR2 antibody and analyzed by flow cytometry (see Methods). The gate was set according to the negative control in the different cell lines. Data are representative of at least four independent experiments.

**Supplementary Fig. 3** **Subtype-specific expression and regulation of Shp1 and CXCR2 in breast cancer cell lines.**

*A.* Representative western blot using anti-phosphoserine 591 in Shp1 (pSer591-Shp1) in MCF10A cells treated with PBS (vehicle control; -) or IL8 (250 ng/ml; +) for 1 h. Total Shp1 and GAPDH were used as loading controls. Data are representative of at least three independent experiments. Molecular weight standards (kDa) are indicated on the left of each panel. *B.* Representative western blot using anti-CXCR2 specific antibody in T47D cells treated with PBS (vehicle control; -) or SSG (10 μM) for 48 h. β−tubulin was used as loading control. Data are representative of three independent experiments. Molecular weight standards (kDa) are indicated on the left of each panel. *C*. Densitometric quantification of the blot in B. **P< 0.005 versus control cells (Student’s t-tests).

*D.* Analysis by qRT-PCR of mRNA levels of Shp1 in a panel of breast cancer cell lines (MCF7, T47D, SK-BR-3, MDA-MB-453, MDA-MB-231, BT-549) and non-malignant MCF10A cells. GAPDH was used as housekeeping gene. Data are means ± SD of two independent experiments. *E.* Western blot analysis of Shp1 expression in the same panel of cell lines shown in panel D. Shp1 staining is shown with two different exposure times (low and high) for better interpretation. Actin and ponceau staining were used as loading controls. *F and G*. Volcano plots for comparison ‘luminal vs HER2’ cell lines (F) and ‘TNBC vs HER2’ cell lines (G), showing significance (as -log_10_ transformed p-value) against magnitude (log_2_ (fold change)). Genes identified as significantly differentially expressed are represented as red (up-regulated) or blue (down-regulated) dots. Vertical and horizontal lines represent the fold change and p-value thresholds applied, respectively.

| **Subtype** | **Cell line** | **GEO accession (GSM ID)** |
| --- | --- | --- |
| Luminal / ER+ | ZR-75-1 | GSM1401648 |
|  | BT-474 | GSM1401649 |
|  | MCF-7 | GSM1401653 |
|  | T-47-D | GSM1401654 |
|  | MDA-MB-361 | GSM1401664 |
|  | ZR-75-30 | GSM1401666 |
| HER2-positive | SK-BR-3 | GSM1401659 |
|  | HCC-1954 | GSM1401665 |
|  | MDA-MB-453 | GSM1401667 |
|  | BT-20 | GSM1401673 |
|  | HCC-1569 | GSM1401674 |
| Triple-negative (TNBC) | MDA-MB-468 | GSM1401650 |
|  | HCC-38 | GSM1401651 |
|  | SUM-159 | GSM1401652 |
|  | HCC-1599 | GSM1401655 |
|  | MDA-MB-436 | GSM1401656 |
|  | BT-549 | GSM1401657 |
|  | MDA-MB-231 | GSM1401658 |
|  | HCC-70 | GSM1401660 |
|  | HCC-1143 | GSM1401661 |
|  | MDA-MB-134 | GSM1401662 |
|  | HCC-1937 | GSM1401668 |
|  | MDA-MB-157 | GSM1401671 |
|  | HCC-1187 | GSM1401672 |
|  | SUM-102 | GSM1401675 |

**Supplementary Table S1. Breast cancer cell lines included in the transcriptomic dataset GSE58135.** Transcriptomic analyses were performed using the GSE58135 dataset, which profiles gene expression across 25 breast cancer cell lines representing all major molecular subtypes: luminal/ER-positive (n = 6), HER2-positive (n = 5), and triple-negative (n = 14). This dataset provides a heterogeneous and representative panel for comparative evaluation of IL8/CXCR2/Shp1-related signaling pathways across distinct molecular contexts.
