# Supplementary material for: Shp1 phosphatase regulates CXCR2 protein stability and IL8-mediated invasiveness in breast cancer
Source: Cell Death Dis. 2026 Mar 2;17(1):297. doi: 10.1038/s41419-026-08516-4 (PMC13039404; doi:10.1038/s41419-026-08516-4)

Figure 1B

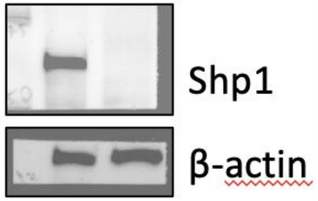

Figure 3 A

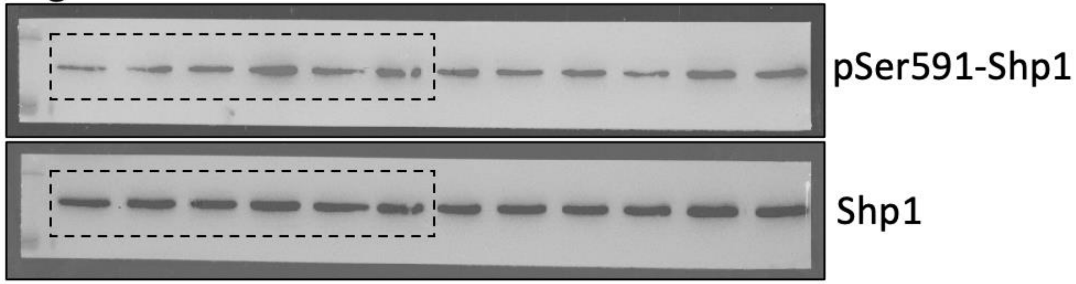

Figure 3 D

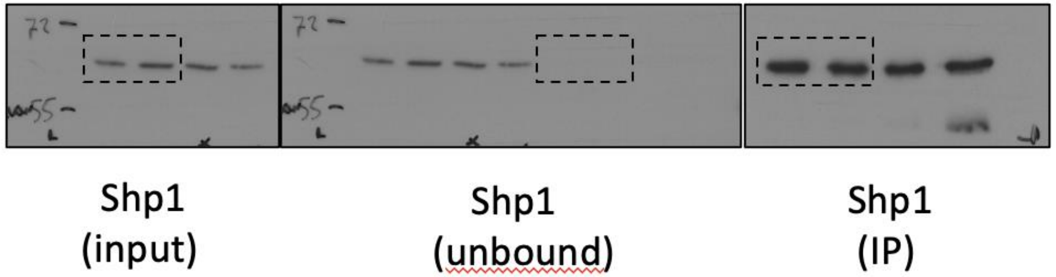

Figure 3 F

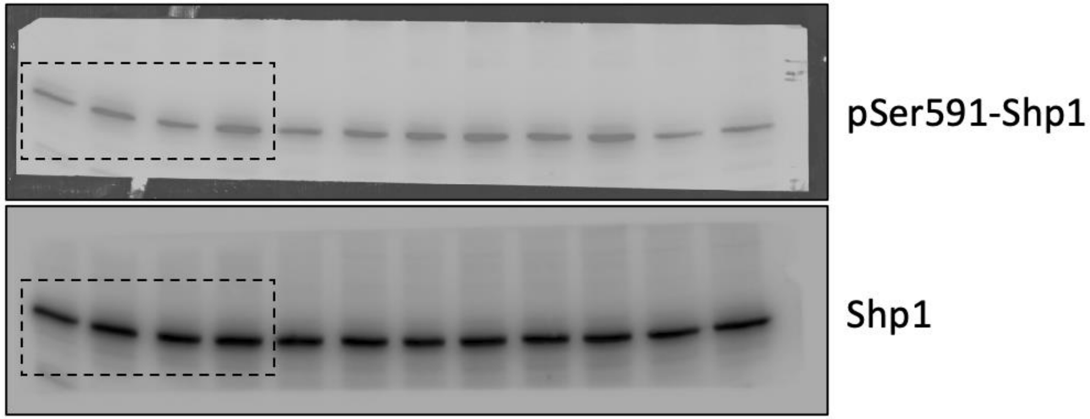

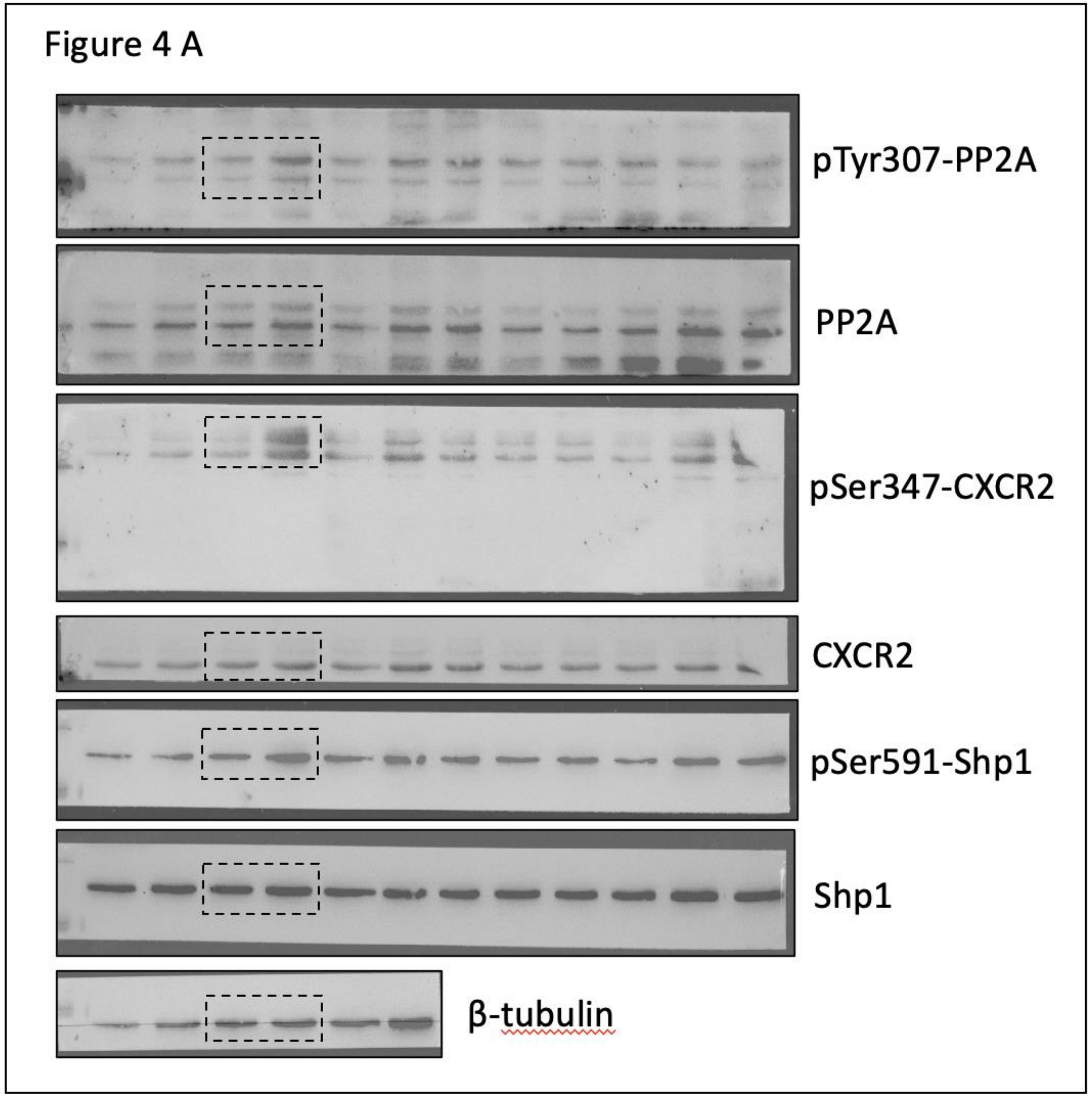
Figure 4 A
pTyr307-PP2A
PP2A
pSer347-CXCR2
CXCR2
pSer591-Shp1
Shp1
β-tubulin

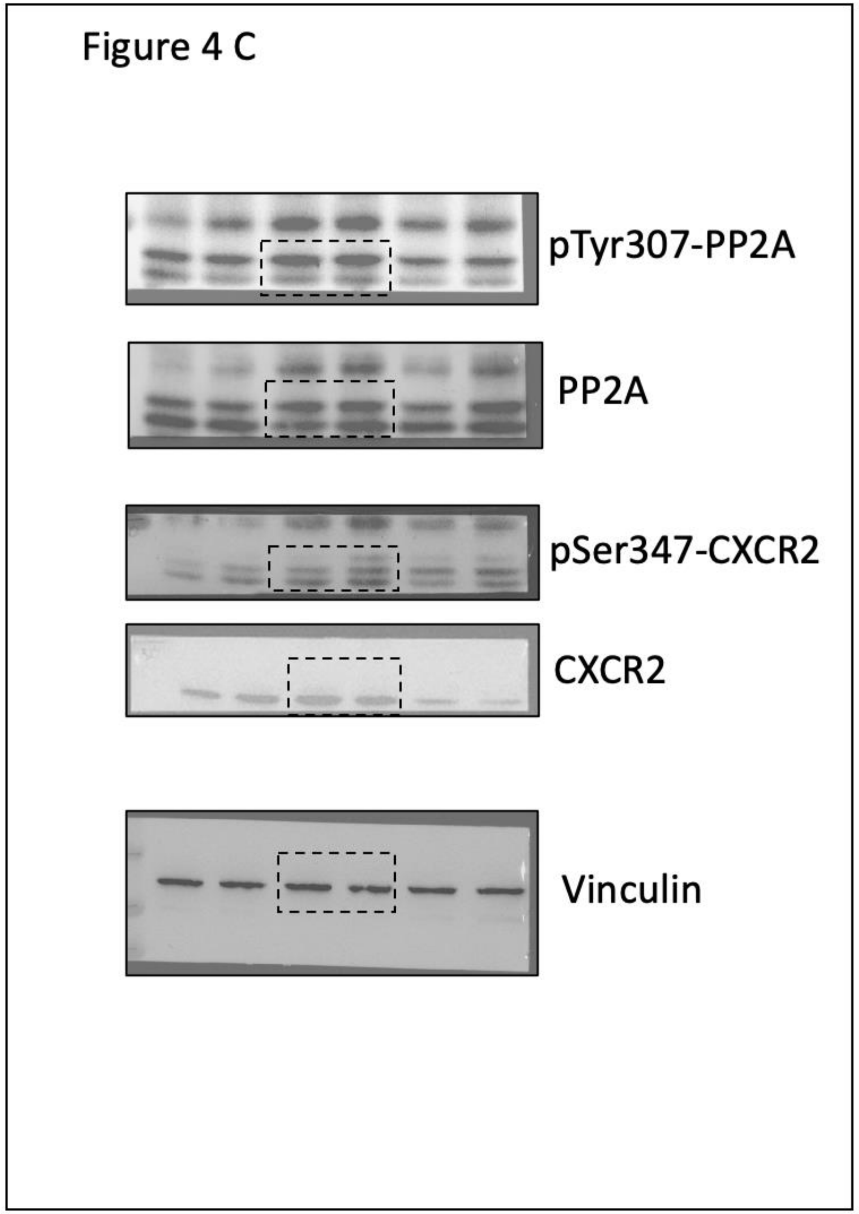
Figure 4 C
pTyr307-PP2A
PP2A
pSer347-CXCR2
CXCR2
Vinculin

Figure 4 D

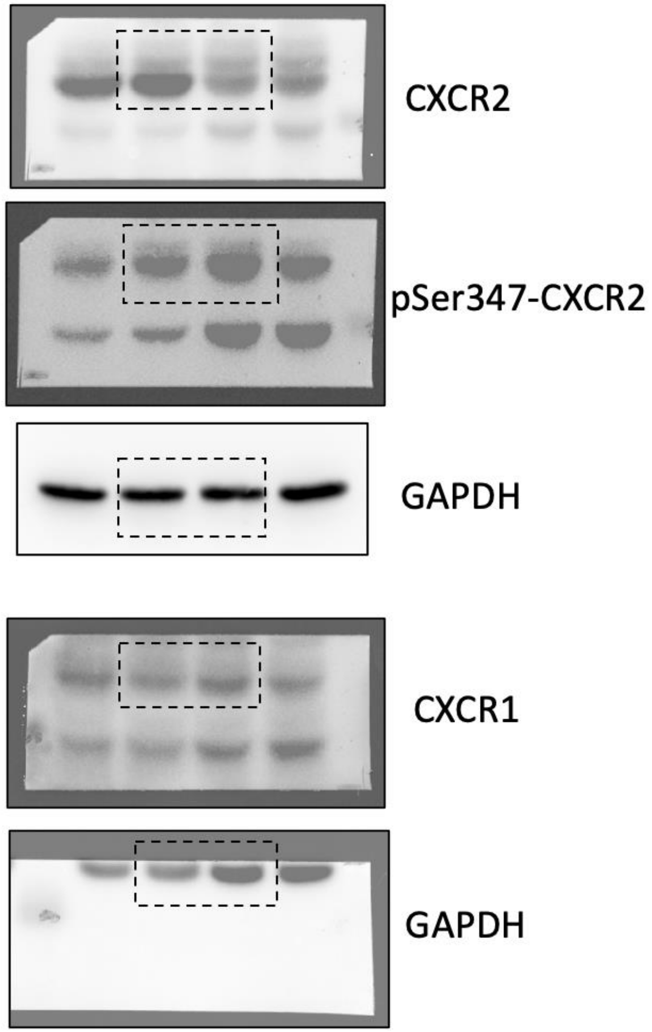

Figure 4 H

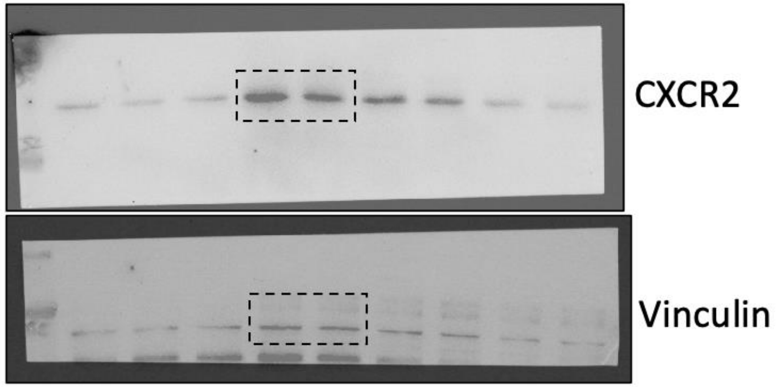

Figure 4 J

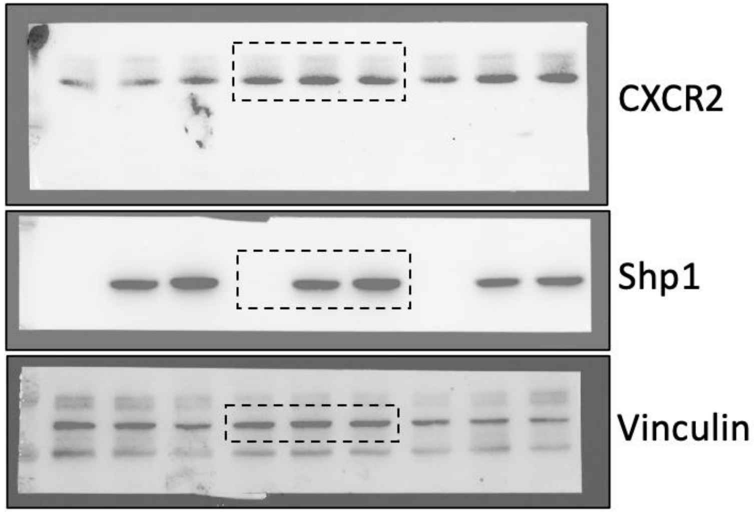

Figure 5 A

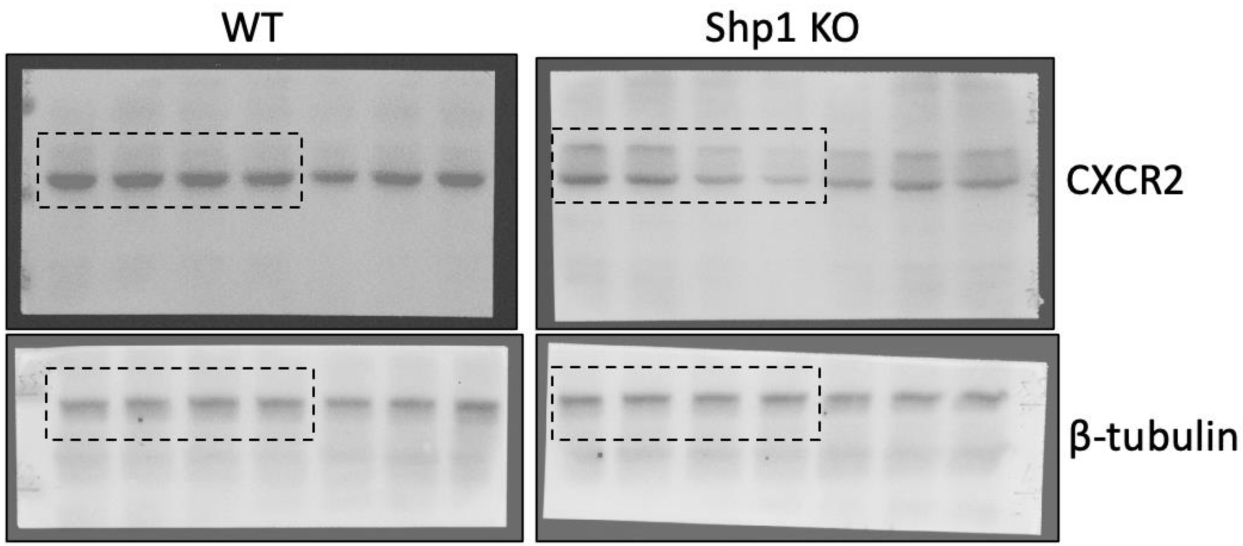

Figure 5 C

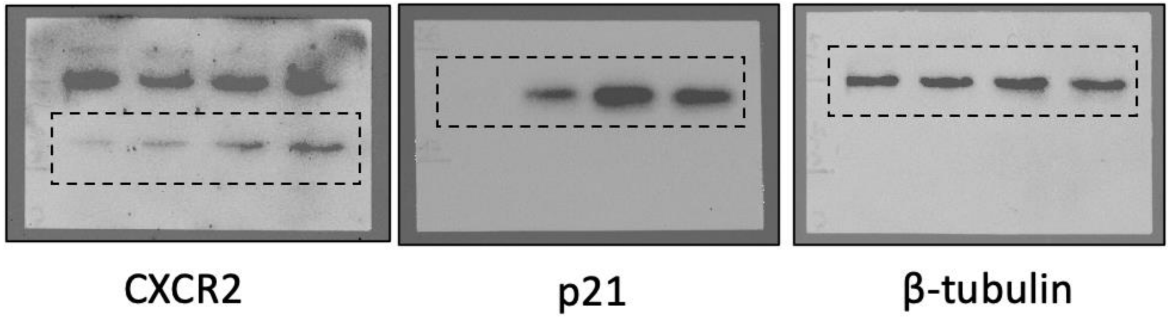

Figure 5 E

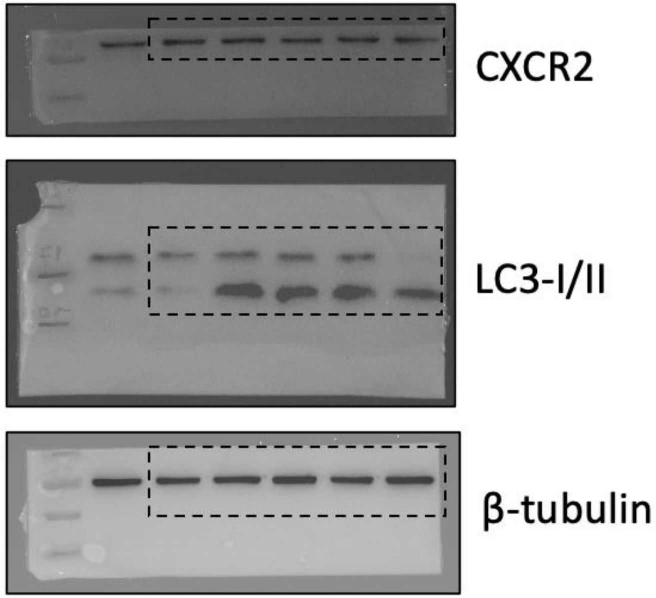

Figure 5 F

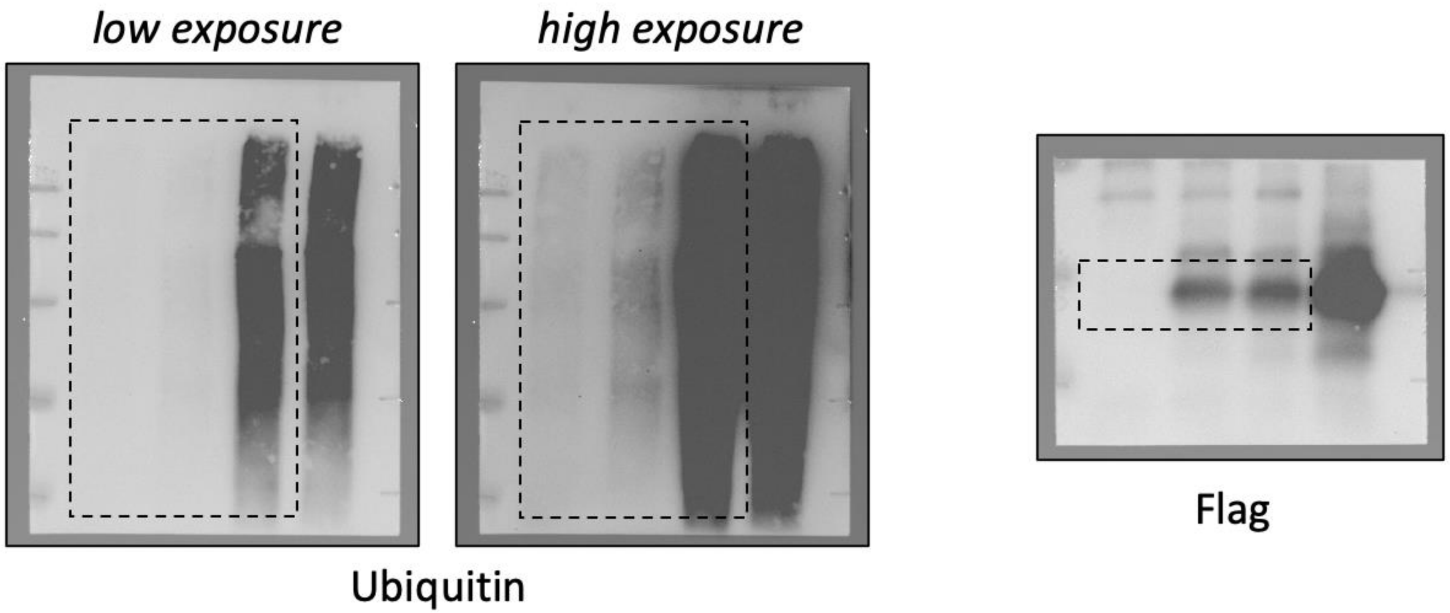

Figure 5 H

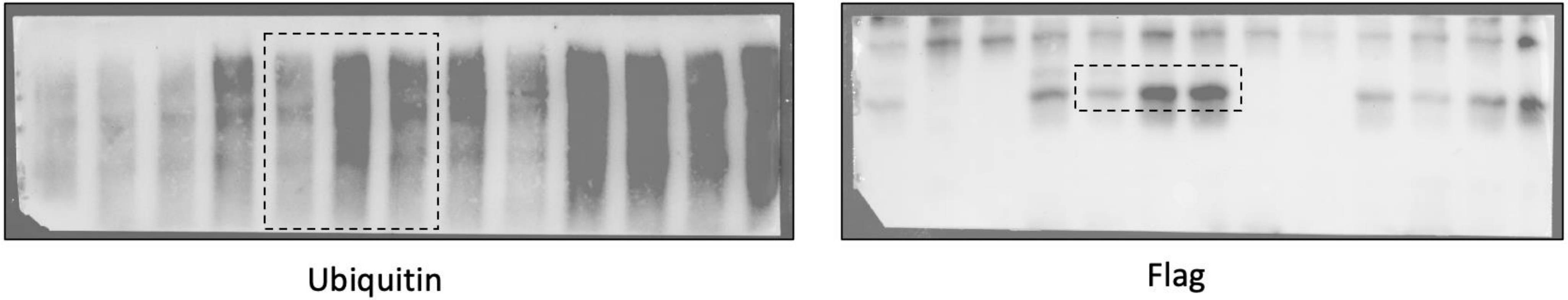

Figure 5 J

BD FACSDiva 8.0.1

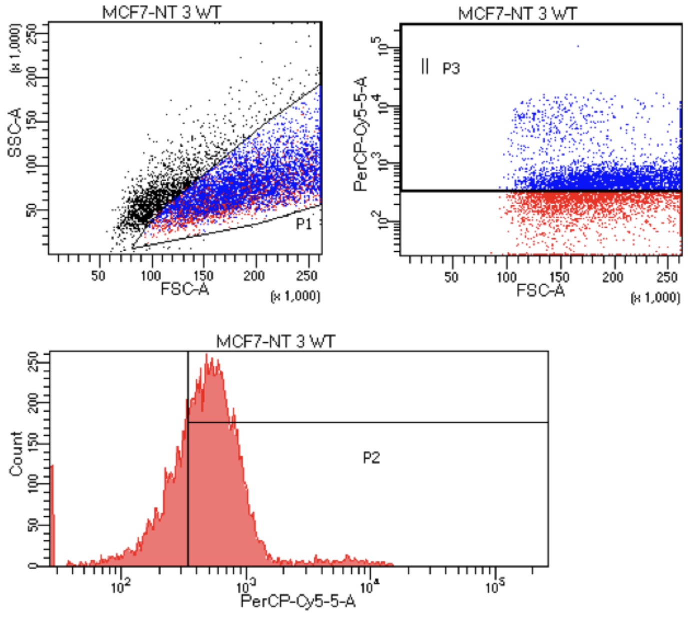

Experiment Name:	13-12-2024
Specimen Name:	MCF7
Tube Name:	NT 3 WT
Record Date:	Dec 13, 2024 11:36:12 AM
SOP:	Administrator
GUID:	4ab30489-371e-4a21-9468-9037b61e...

Population	#Events	%Parent	PerCP-Cy5-5-A Mean
P1	10,000	75.3	754
P2	7,040	70.4	982
P3	7,112	71.1	975

Tube: NT 3 WT

Population	#Events	%Parent	%Total
All Events	13,286	####	100.0
P1	10,000	75.3	75.3
P2	7,040	70.4	53.0
P3	7,112	71.1	53.5

BD FACSDiva 8.0.1

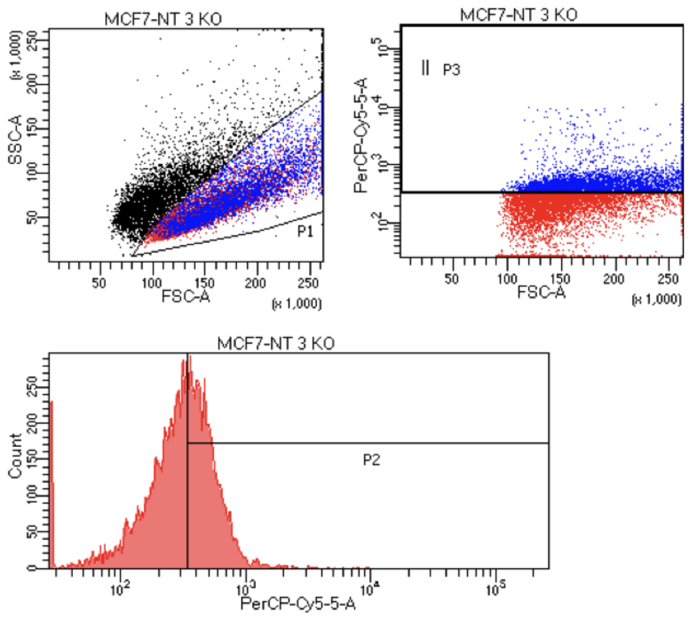

Experiment Name:	2024-10-31
Specimen Name:	MCF7
Tube Name:	NT 3 KO
Record Date:	Oct 31, 2024 3:14:45 PM
SOP:	Administrator
GUID:	409df284-fd14-4690-8d22-52ef88d8e3...

Population	#Events	%Parent	PerCP-Cy5-5-A Mean
P1	10,000	66.8	388
P2	4,400	44.0	626
P3	4,476	44.8	621

Tube: NT 3 KO

Population	#Events	%Parent	%Total
All Events	14,965	####	100.0
P1	10,000	66.8	66.8
P2	4,400	44.0	29.4
P3	4,476	44.8	29.9

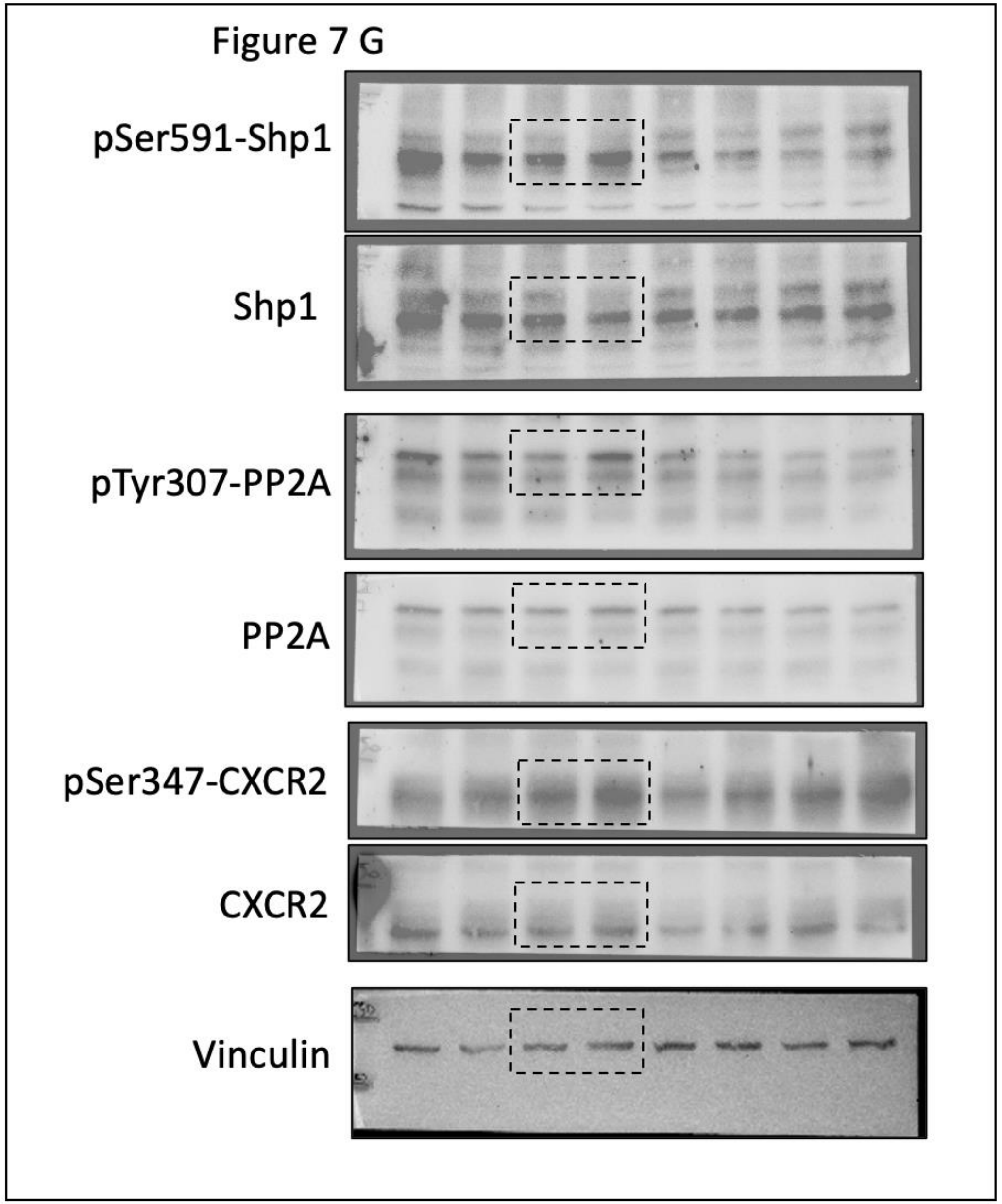
Figure 7 G
pSer591-Shp1
Shp1
pTyr307-PP2A
PP2A
pSer347-CXCR2
CXCR2
Vinculin

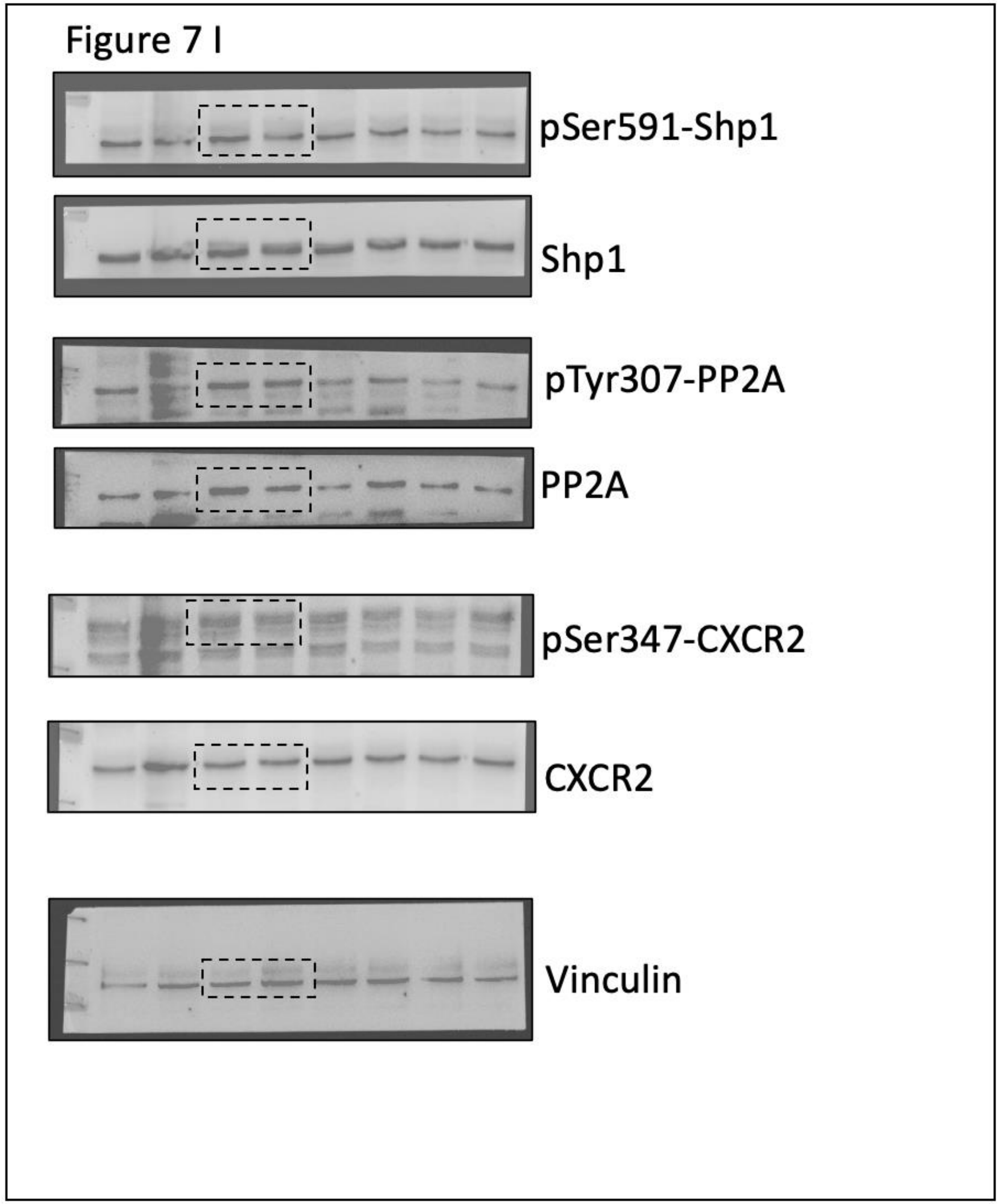
Figure 7 I
pSer591-Shp1
Shp1
pTyr307-PP2A
PP2A
pSer347-CXCR2
CXCR2
Vinculin

Figure 7F

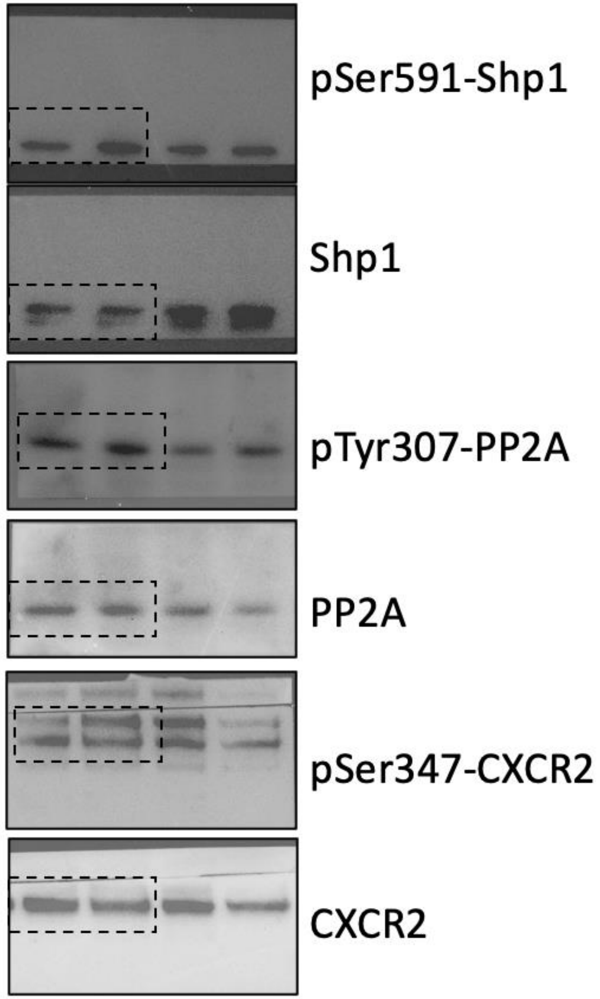

Figure 7H

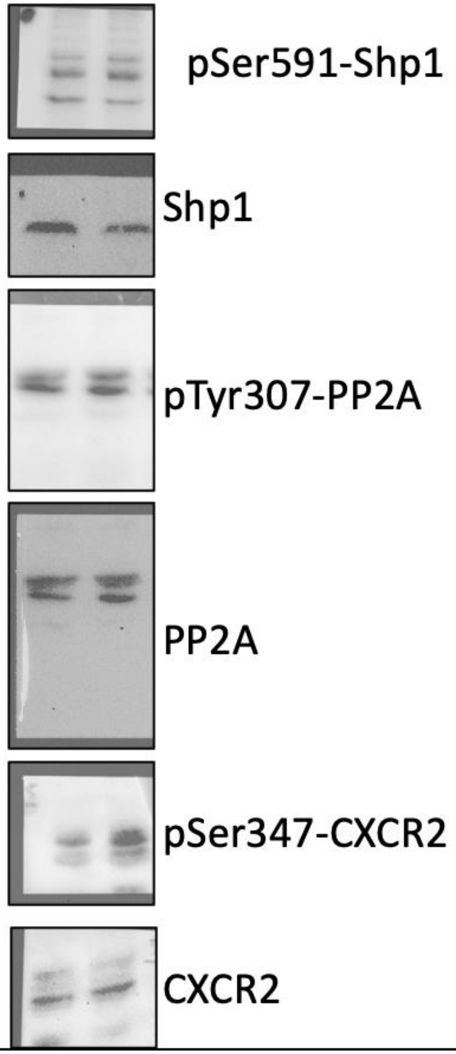

Figure 7J

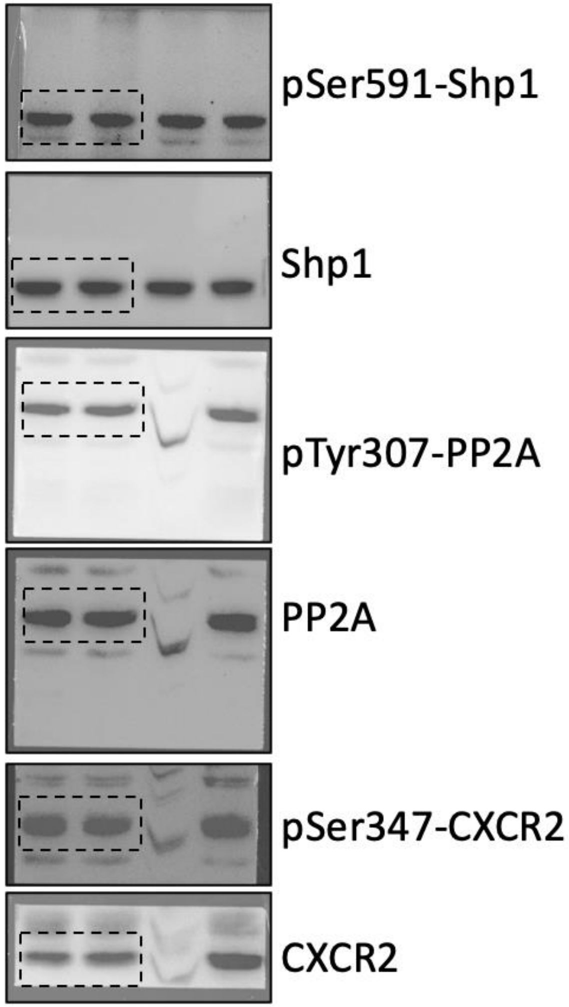

Supplementary Figure 1G

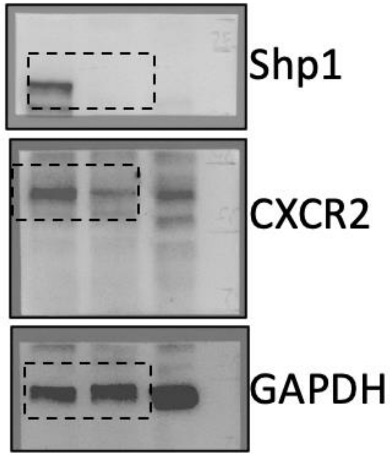

Supplementary Figure 1I

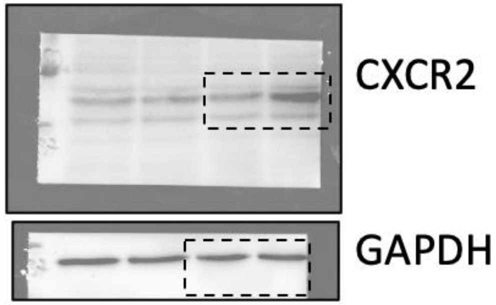

Supplementary Figure 1H

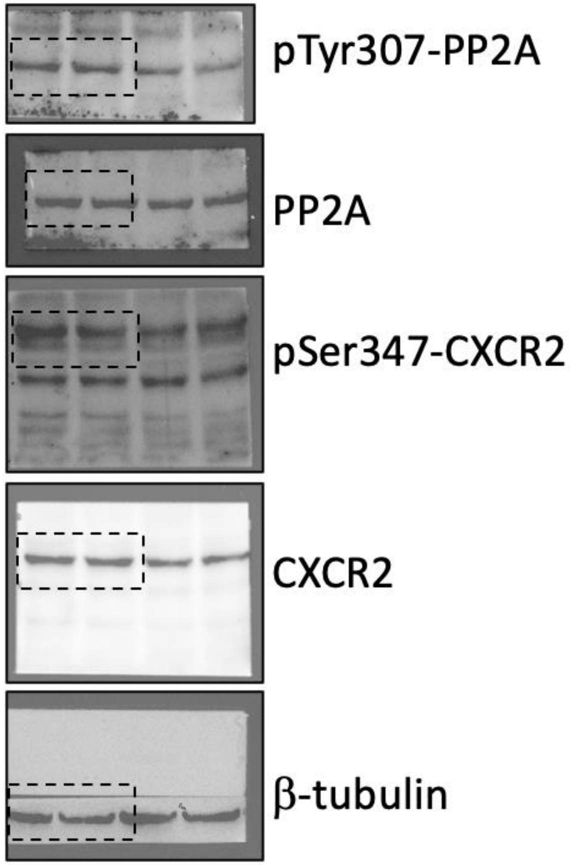

Supplementary Figure 1K

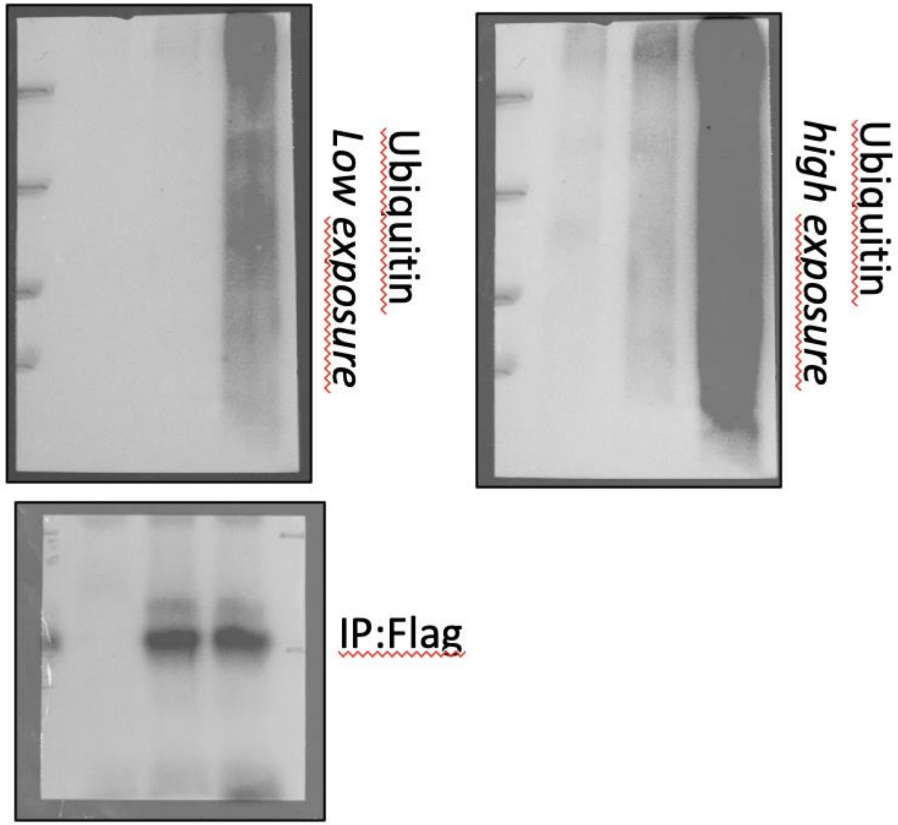

Supplementary Figure 2E

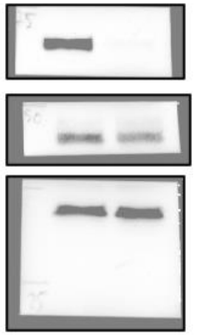

Supplementary Figure 3A

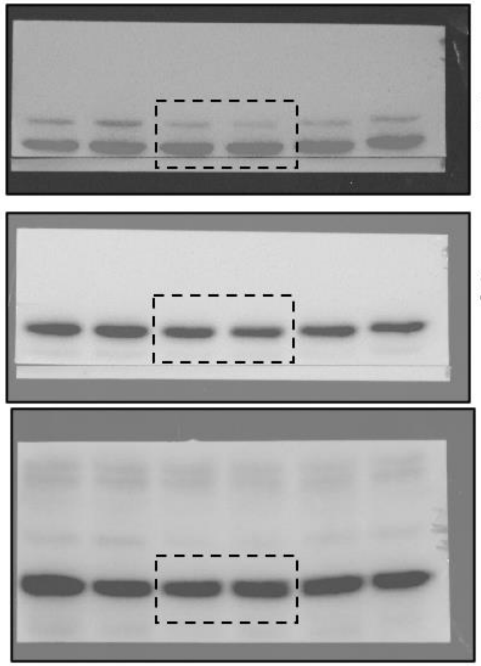

Supplementary Figure 3B

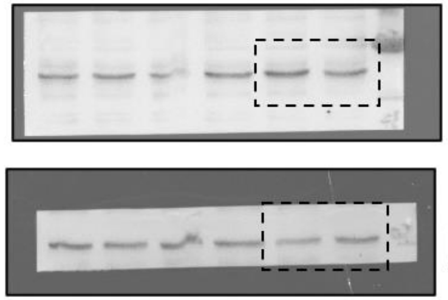

Supplementary Figure 3E

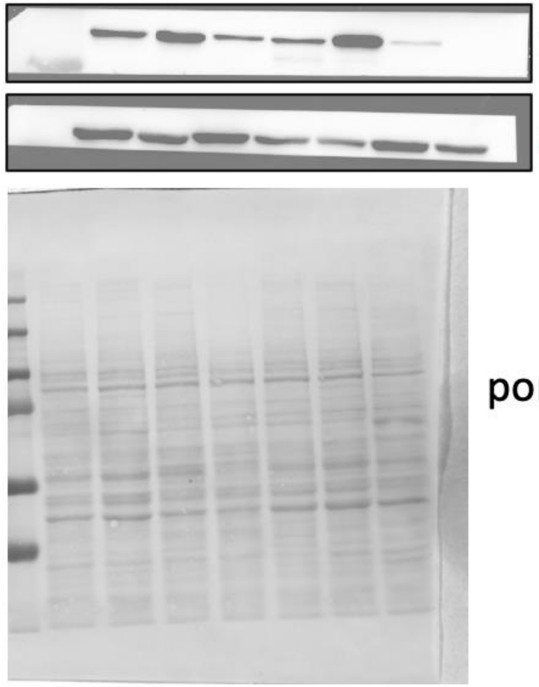

Supplement: Supplementary file 2 — Original Western Blots [file 41419_2026_8516_MOESM2_ESM.pdf]
